# Engineering analysis of multienzyme cascade reactions for 3ʹ‐sialyllactose synthesis

**DOI:** 10.1002/bit.27898

**Published:** 2021-08-02

**Authors:** Sabine Schelch, Manuel Eibinger, Stefanie Gross Belduma, Barbara Petschacher, Jürgen Kuballa, Bernd Nidetzky

**Affiliations:** ^1^ Austrian Centre of Industrial Biotechnology Graz Austria; ^2^ Institute of Biotechnology and Biochemical Engineering Graz University of Technology, NAWI Graz Graz Austria; ^3^ GALAB Laboratories GmbH Hamburg Germany

**Keywords:** 3ʹ‐sialyllactose, α2,3‐sialyltransferase, biocatalysis, CMP‐*N*‐acetyl‐ d‐neuraminic acid, lactose, lyase, multienzyme cascade reaction, *N*‐acetyl‐ d‐mannosamine, *N*‐acetyl‐ d‐neuraminic acid (Neu5Ac), sialo‐oligosaccharides, synthase

## Abstract

Sialo‐oligosaccharides are important products of emerging biotechnology for complex carbohydrates as nutritional ingredients. Cascade bio‐catalysis is central to the development of sialo‐oligosaccharide production systems, based on isolated enzymes or whole cells. Multienzyme transformations have been established for sialo‐oligosaccharide synthesis from expedient substrates, but systematic engineering analysis for the optimization of such transformations is lacking. Here, we show a mathematical modeling‐guided approach to 3ʹ‐sialyllactose (3SL) synthesis from *N*‐acetyl‐ d‐neuraminic acid (Neu5Ac) and lactose in the presence of cytidine 5ʹ‐triphosphate, via the reactions of cytidine 5ʹ‐monophosphate‐Neu5Ac synthetase and α2,3‐sialyltransferase. The Neu5Ac was synthesized in situ from *N*‐acetyl‐ d‐mannosamine using the reversible reaction with pyruvate by Neu5Ac lyase or the effectively irreversible reaction with phosphoenolpyruvate by Neu5Ac synthase. We show through comprehensive time‐course study by experiment and modeling that, due to kinetic rather than thermodynamic advantages of the synthase reaction, the 3SL yield was increased (up to 75%; 10.4 g/L) and the initial productivity doubled (15 g/L/h), compared with synthesis based on the lyase reaction. We further show model‐based optimization to minimize the total loading of protein (saving: up to 43%) while maintaining a suitable ratio of the individual enzyme activities to achieve 3SL target yield (61%–75%; 7–10 g/L) and overall productivity (3–5 g/L/h). Collectively, our results reveal the principal factors of enzyme cascade efficiency for 3SL synthesis and highlight the important role of engineering analysis to make multienzyme‐catalyzed transformations fit for oligosaccharide production.

## Graphical abstract

Sialo‐oligosaccharides are important carbohydrate products and multienzyme transformations enable their production. Here, the authors developed a modeling‐guided approach for engineering analysis and optimization of 3'‐sialyllactose (3SL) synthesis. Intermediary N‐acetyl‐D‐neuraminic acid (Neu5Ac) was obtained from N‐acetyl‐D‐mannose (ManNAc) with pyruvate (PYR) or phosphoenolpyruvate (PEP) using lyase (NAL) or synthase (SiaC), respectively. Neu5Ac was activated by synthetase (CSS) and transferred to lactose catalyzed by a2,3‐sialyltransferase (PdST). Guided by the model, the total enzyme loading was minimized, while retaining target yield at high productivity.

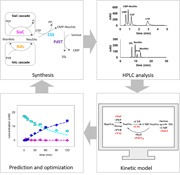

**Abbreviations**
3SL: 3ʹ‐sialyllactose
CIP: calf intestine alkaline phosphatase
CMP: cytidine 5ʹ‐monophosphate
CSS: CMP‐sialic acid synthetase (EC 2.7.7.43)
CTP: cytidine 5ʹ‐triphosphate
ManNAc: *N*‐acetyl‐d‐mannosamine
NAL: Neu5Ac lyase (EC 4.1.3.3)
Neu5Ac: *N*‐acetyl‐d‐neuraminic acid
PdST: α2,3‐sialyltransferase from *P. dagmatis* (EC 2.4.99.‐)
PEP: phosphoenolpyruvate
PYR: pyruvate
SiaC: sialic acid synthase (EC 2.5.1.56)

## 1 INTRODUCTION

Sialo‐oligosaccharides have received increased attention as health‐promoting ingredients for food and feed use (Faijes et al., 2019; Lu et al., 2021). Their synthesis as industrial products (e.g., 3ʹ‐sialyllactose, 3SL) drives an emerging biotechnology for the mass production of structurally defined, complex carbohydrates (Bode et al., 2016). 3SL is one of simplest of the human milk oligosaccharides (X. Chen, 2015) and is currently considered strongly for commercial use in infant formula (Bych et al., 2019). Core task of every sialo‐oligosaccharide process is to provide from expedient substrates the naturally scarce sialic acid (e.g., *N*‐acetyl‐d‐neuraminic acid, Neu5Ac) in a form usable for enzymatic glycosylation (Fessner, 2015). The glycosylation involves stereo‐ and regioselective transfer of the sialic acid to the acceptor oligosaccharide (e.g., lactose) (R. Chen, 2015; Na et al., 2021; Weijers et al., 2008). It is typically catalyzed by a sialyltransferase (EC 2.4.99.‐). The enzyme uses a cytidine 5ʹ‐monophosphate (CMP)‐activated sialic acid (e.g., CMP‐Neu5Ac) as the substrate which for reason of process cost effectiveness cannot be added as a reagent, but must be synthesized directly in the reaction (Li et al., 2019). Bio‐catalysis in multienzyme cascades, integrating glycosylation with supply of CMP‐Neu5Ac, is therefore central to the development of sialo‐oligosaccharide production systems, irrespective of whether isolated enzymes or live whole cells are used (Schelch et al., 2020). Multienzyme transformations have been established for sialo‐oligosaccharide synthesis with both systems (Schelch et al., 2020), but systematic engineering analysis of the reaction cascades for the optimization of such transformations is lacking. Kinetic modeling‐based approaches are important engineering tools for coping with the inherent complexity of enzymatic cascades for efficient process development (Kitamura et al., 2020; Schmideder et al., 2017; Xue & Woodley, 2012; Zhong et al., 2017; Zimmermann et al., 2007). However, use of kinetic modeling for studying the assembly of oligosaccharides by sugar nucleotide‐dependent transferases is generally scarce (Mahour et al., 2018; Rexer et al., 2017) and is missing entirely with sialo‐oligosaccharides. Fundamental engineering problems, like the identification of main bottlenecks of conversion efficiency of sialo‐oligosaccharide‐producing enzyme cascade reactions, thus remain largely unaddressed. Relevant progress is important to advance the biocatalytic synthesis by using these enzymatic systems. It furthermore provides essential guidance to metabolic engineering efforts in cell factory development (e.g., 3SL‐producing *Escherichia coli*; Faijes et al., 2019; Lu et al., 2021).

**Figure 1:**
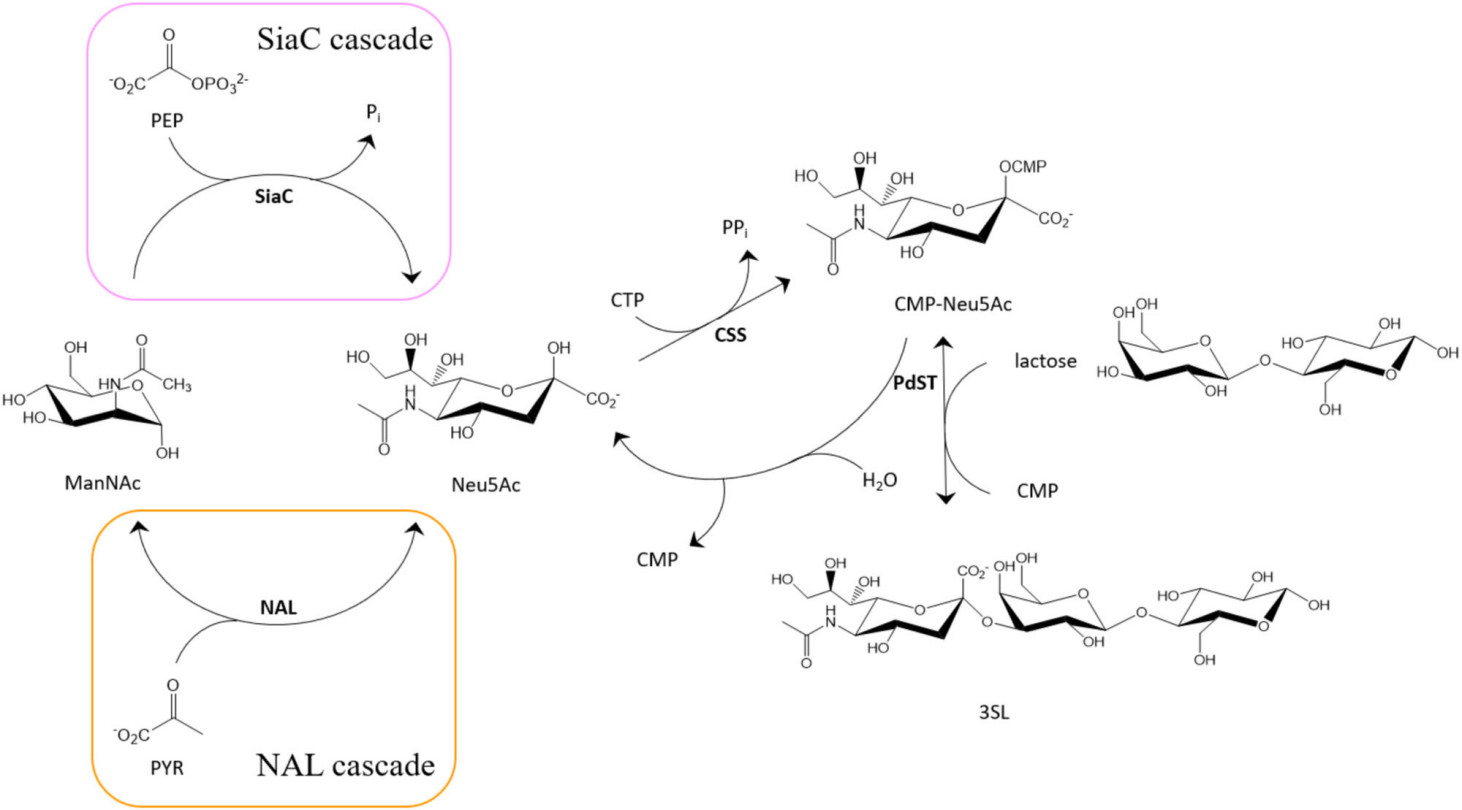
3SL synthesis from ManNAc in enzymatic cascade transformations. Hydrolysis of CMP‐Neu5Ac by PdST is shown. 3SL, 3ʹ‐sialyllactose; CMP‐Neu5Ac, cytidine 5ʹ‐monophosphate‐*N*‐acetyl‐d‐neuraminic acid; ManNAc, *N*‐acetyl‐d‐mannosamine; PdST, α2,3‐sialyltransferase from *Pasteurella dagmatis*

As shown in Figure 1, we here consider the common, three‐step “core route” to sialo‐oligosaccharides, involving formation of CMP‐Neu5Ac from *N*‐acetyl‐d‐mannosamine (ManNAc), pyruvate (PYR) or phosphoenolpyruvate (PEP), and CTP (Li et al., 2017; Tasnima et al., 2019; Yu et al., 2006). The condensation of ManNAc and PYR is catalyzed by Neu5Ac lyase (NAL; EC 4.1.3.3) and the herein used NAL is from *Lactobacillus plantarum* WCFS1 (Sánchez‐Carrón et al., 2011). Biocatalytic production of Neu5Ac by NAL, however, uncoupled from sialoside synthesis, was examined in several earlier studies (Blayer et al., 1999; Kragl et al., 1991; Mahmoudian et al., 1997; Maru et al., 1998; Tao et al., 2011), that also included modeling efforts (Klermund et al., 2016; Zimmermann et al., 2007). Considering the relatively high *K*_M_ for ManNAc of this (~160 mM) and other NAL enzymes (Schelch et al., 2020), we examine an alternative cascade reaction in which sialic acid synthase (SiaC; EC 2.5.1.56) is used. The SiaC requires PEP instead of PYR as the substrate and shows a lower *K*_M_ for ManNAc (9.4 mM) than NAL. The SiaC from *Neisseria meningitidis* is used here (Gunawan et al., 2005; Hao et al., 2005). Besides kinetic advantages, the SiaC reaction has an equilibrium far on the Neu5Ac side, driven by the PEP conversion. The NAL reaction is reversible and has an unfavorable reaction equilibrium (Groher & Hoelsch, 2012). Although used before in metabolic engineering of *Escherichia coli* for sialo‐oligosaccharide production (Fierfort & Samain, 2008), the SiaC reaction was never applied in corresponding cascade reactions done with isolated enzymes in vitro. Conversion of Neu5Ac into CMP‐Neu5Ac is catalyzed by CMP‐sialic acid synthetase (CSS; EC 2.7.7.43) and requires cytidine 5ʹ‐triphosphate (CTP). The CSS from *N. meningitidis* is used here, for the comparably high specific activity it has among the reported enzymes of this class (Gilbert et al., 1997; He et al., 2011). To obtain 3SL, the sialylation of lactose is catalyzed by the α2,3‐sialyltransferase from *Pasteurella dagmatis* (PdST, Schmölzer et al. 2013, 2015). Besides the sialyl‐transfer which yields 3SL, PdST has a distinctive hydrolase activity against CMP‐Neu5Ac (Figure 1). While the presence of acceptor substrate reduces this hydrolase activity (Schmölzer et al., 2017), it remains a significant side reaction. PdST can also act as a sialidase. The sialidase reaction, which overall releases Neu5Ac and lactose from 3SL, requires CMP (Mehr & Withers, 2016). It is believed to proceed via intermediary CMP‐Neu5Ac formed through the reverse transferase reaction (3SL + CMP → CMP‐Neu5Ac + lactose) (Mehr & Withers, 2016). The CMP‐Neu5Ac is then hydrolyzed. The sialidase activity of PdST is found low when assayed directly from 3SL (Schmölzer et al., 2013) but its possible role in a synthesis reaction cannot be excluded.

In this study, we performed systematic engineering analysis for 3SL synthesis by NAL/CSS/PdST and SiaC/CSS/PdST cascade reactions. Through quantification of all relevant reactants from the enzymatic conversions, we obtained detailed time‐course data for the multistep transformations and used them for kinetic model development. A mass action‐based, Michaelis–Menten type kinetic model (Bulik et al., 2009) was used to describe the complete “reaction dynamics” in 3SL syntheses by the two enzymatic systems. Due to kinetic advantages (*K*_M_ for ManNAc) of the SiaC compared with the NAL reaction, the 3SL productivity was generally enhanced when the SiaC/CSS/PdST system was used. The validated Michaelis–Menten model was employed for facile reaction “optimization” through an innovative in silico approach. A constrained random simulation of a large set of reaction conditions (≥10^5^) with variable enzyme ratios was carried out. From the results of this high‐throughput computational sampling, as a typical objective for the optimization of enzyme cascade reactions, conditions were selected that involved the least usage of total enzyme to satisfy a given conversion task. The model‐predicted optima were verified experimentally. Collectively, our results demonstrate kinetic modeling‐guided development of efficient enzyme cascades for 3SL synthesis. Our findings highlight the important role of such analysis in the optimization of multienzyme biocatalysts for oligosaccharide production.

## 2 MATERIALS AND METHODS

### 2.1 Materials

ManNAc, CTP (95% purity; 5% CDP) and CMP (both disodium salts), CMP‐Neu5Ac, Neu5Ac, and 3SL were from Carbosynth (Compton, Berkshire, UK). PYR, PEP (both sodium salts), 2‐nitrophenyl‐β‐d‐galactopyranoside (oNP‐Gal) and lactose (monohydrate) were from Sigma Aldrich/Fluka (Vienna, Austria). Q5® High‐Fidelity DNA polymerase, dNTPs, calf intestine alkaline phosphatase (CIP) were from New England Biolabs (Frankfurt am Main, Germany). All other chemicals were of reagent grade from Sigma Aldrich/Fluka or Roth (Karlsruhe, Germany).

### 2.2 Enzyme expression and purification

#### 2.2.1 Strains, plasmids, and media

*E. coli* BL21 and *E. coli* BL21 (DE3) were used. Genes for *N. meningitidis* CSS (UniProtKB ‐ Q7DDU0) and SiaC (UniProtKB ‐ P0A0Z8) were from Galab Laboratories. Both were cloned (Online Supporting Information) into a pC21e1 expression vector reported recently (Zhong et al., 2019). The NAL gene from *L. plantarum* WCFS1 (GenBank CCC80530.1) was codon‐optimized for expression in *E. coli* and received in a pET22b (+) vector (GenScript Biotech). The gene for ManNAc 1‐dehydrogenase (ManNAcDH, E.C. 1.1.1.233) from *Flavobacterium* sp. 141‐8 was kindly provided in a pET‐28a(+)‐vector by Kathrin Castiglione (Friedrich‐Alexander Universität Erlangen‐Nürnberg, Germany) (Klermund et al., 2016). *E. coli* strains were cultured in LB broth and agar plates.

#### 2.2.2 Expression and purification of enzymes

PdST was obtained as described by Schmölzer et al. (2013). Expression was done similarly for all enzymes (1 mM isopropyl β‐d‐thiogalactopyranoside; 20 h) except that 25°C (SiaC, NAL, and ManNAcDH) or 37°C (CSS) was used during induction. Purification was done by His‐tag affinity chromatography (Online Supporting Information). Enzyme purity was verified by sodium dodecyl sulfate‐polyacrylamide gel electrophoresis (Figure S1). Protein was determined with Roti‐Quant reagent (Roth) referenced to bovine serum albumin (BSA). Enzyme stock solutions were stored (~20 mg/ml; buffer) at −20°C without loss of activity for at least 21 days.

#### 2.2.3 Activity assays

Assays were conducted in duplicate in 200 µl total volume of 100 mM Tris/HCl buffer, pH 8.0, containing 1 mg/ml BSA. Temperature (37°C) and agitation rate (450 rpm) were controlled in a Thermomixer comfort (Eppendorf). The CSS reaction contained 5 mM Neu5Ac, 25 mM CTP, 20 mM MgCl_2_, 0.2 mM l‐cysteine, and 0.1 µM enzyme. The PdST reaction (Schmölzer et al., 2014) contained 1 mM CMP‐Neu5Ac, 1 mM oNP‐Gal, and 0.1 µM enzyme. At certain times, ice‐cold acetonitrile (20 µl) was added to 20 µl of reaction sample and incubation continued on ice for 15 min. Samples were analyzed by ion‐pairing high‐performance liquid chromatography (HPLC) as described below (Section 2.3.1). The SiaC reaction contained 20 mM ManNAc, 20 mM PEP, 20 mM MgCl_2_, and 0.2 µM enzyme. The NAL reaction mixture contained 20 mM ManNAc, 50 mM PYR, and 1 µM enzyme. Samples (20 µl) were heated (99°C, 15 min) using a Thermomixer, kept on ice for 15 min, and analyzed enzymatically for ManNAc (see Section 2.3.2) or by carbohydrate HPLC (see Section 2.3.1).

### 2.3 Analytics

#### 2.3.1 HPLC analysis

ManNAc, Neu5Ac, and 3SL were analyzed with a BioRad Aminex® HPX‐87H column on a LaChrom Merck Hitachi system equipped with a Merck Hitachi L‐7400 UV Detector (210 nm) (ligand‐exchange HPLC). Elution was with 5 mM H_2_SO_4_ at 65°C and 0.5 ml/min flow rate. CTP, CMP‐Neu5Ac, CDP, CMP, and cytidine were analyzed on a Shimadzu SPD‐20A system equipped with a Kinetex® 5 µm C‐18 (100 Å; 50 × 4.6 mm) column (ion‐pairing HPLC). Gradient from 6.5% to 25% acetonitrile in 20 mM potassium phosphate buffer (pH 5.9), supplemented with 40 mM tetra‐*n*‐butylammonium bromide as ion‐pairing reagent, was used. The flow rate was 1.5 ml/min at 40°C and detection was at 254 nm.

#### 2.3.2 Enzymatic assay for ManNAc

This was adapted from Klermund et al. (2016) and performed in 96‐well microtiter plates. Sample (20 µl) from NAL or SiaC reaction was added to 145 µl of 100 mM Tris/HCl buffer (pH 8.0). NAD^+^ (1 mM; 20 µl) and ManNAcDH (20 µl; 0.7 mg/ml) were added and incubation done for 20 min at 30°C and 450 rpm. NADH was measured at 340 nm using a multimode microplate reader (BMG Labtech FLUOstar Omega). The ManNAcDH forms one NADH for each ManNAc oxidized. Activity of NAL or SiaC was determined from the ManNAc consumed over time.

### 2.4 Cascade reactions for 3SL synthesis

**Figure 2:**
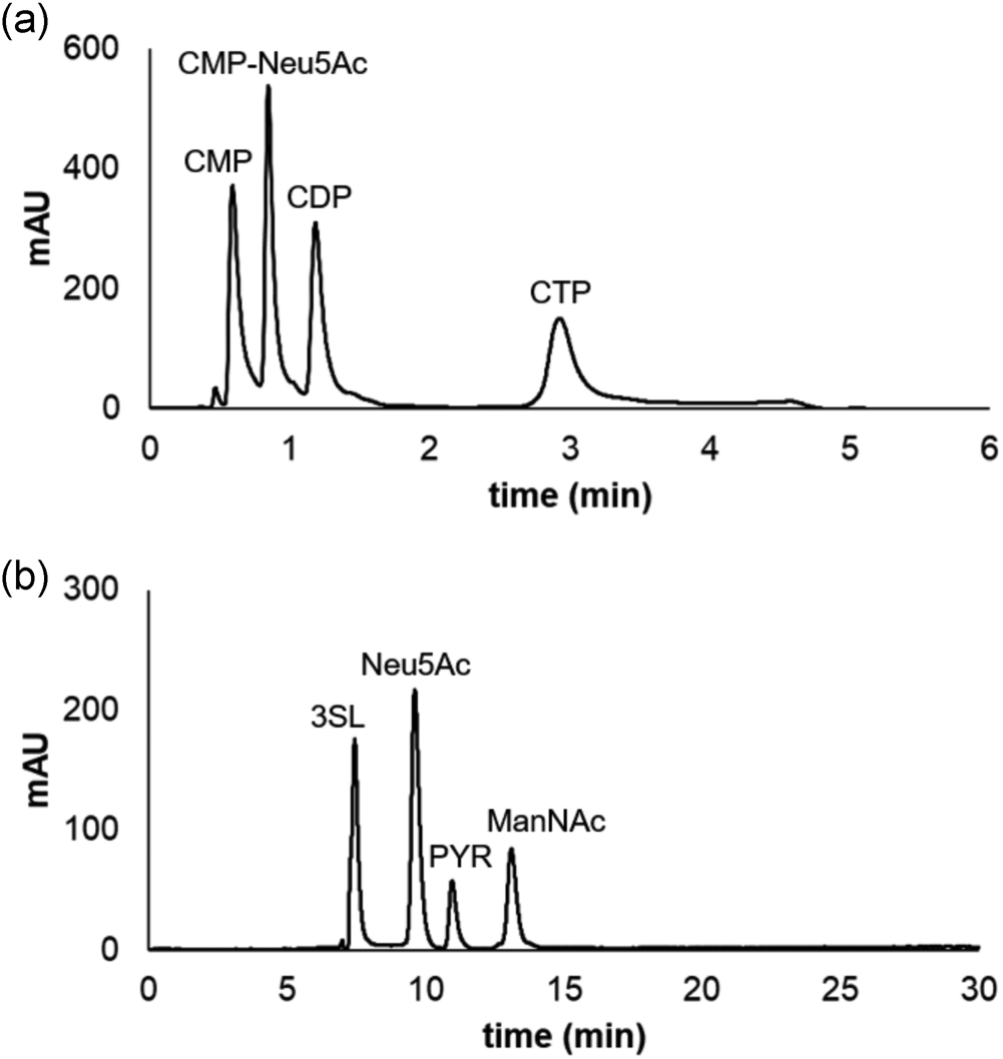
Representative HPLC traces from the analysis of reaction samples performed. (a) Reversed‐phase ion‐pairing HPLC. The CDP (~5%) is from the commercial CTP reagent. (b) Ligand‐exchange HPLC. PEP was converted to PYR by incubation with CIP. For details, see Section 2. CIP, calf intestine alkaline phosphatase; CTP, cytidine 5ʹ‐triphosphate; HPLC, high‐performance liquid chromatography; PEP, phosphoenolpyruvate; PYR, pyruvate

Reactions were done at 37°C and 450 rpm in 1 ml total volume of 100 mM Tris/HCl buffer (pH 8.0), supplemented with 20 mM MgCl_2_ and 0.2 mM l‐cysteine. The NAL/CSS/PdST reactions used 20 mM ManNAc, 50 mM PYR, 25 mM CTP, and 20 mM lactose. The SiaC/CSS/PdST reactions used 20 mM PEP instead of PYR. Sampling involved taking two times 20 µl for analysis by ligand‐exchange and ion‐paring HPLC (Figure 2). The ligand‐exchange sample was heated as described for NAL/SiaC assays and incubated with CIP to hydrolyze the nucleotides (CTP and CMP) present. For this, 15 µl sample received 15 µl MgCl_2_ (200 mM), 15 µl CIP (100 U/ml), and 115 µl Tris/HCl buffer (100 mM, pH 8.0). Incubation was at 30°C and 450 rpm for 4 h. After heating (99°C, 15 min), the chilled CIP reaction was centrifuged (30 min, 13200 rpm) and analyzed by carbohydrate HPLC. To the second reaction sample 20 µl of ice‐cold acetonitrile were added and after keeping the sample on ice for 15 min, it was analyzed by ion‐pairing HPLC.

### 2.5 Kinetic modeling

All modeling was done with MATLAB (R2018).

#### 2.5.1 Model building

The cascade reactions (Figure 1) were translated into the corresponding set of coupled differential equations based on mass balance. Individual reactions (NAL, SiaC, CSS, and PdST) were described by Michaelis–Menten parameter (*V*_max_ and *K*_M_) mass action kinetics, as shown generally in Equation (1) and in full detail in Online Supporting Information.

(1) $$\;V=\frac{V_{\max}[E][S_{1}][S_{2}]\left(1-\frac{\Gamma }{K_{\mathrm{eq}}}\right)}{[S_{1}][S_{2}]+K_{M(S2)}[S_{1}]+K_{M(S1)}[S_{2}]+K_{M(S1)}K_{M(S2)}}\;$$

*V*_max_ is the maximum specific rate, modeled as mmol/(mg enzyme × min) and with values taken from this study or literature (Gilbert et al., 1997; Groher & Hoelsch, 2012; Gunawan et al., 2005; He et al., 2011; Sánchez‐Carrón et al., 2011; Schmölzer et al. 2013, 2015). The overall rate (*V*) in mmol/(L × min) is then given as *V* = *V*_max_ [E], where [E] is the enzyme protein concentration (mg/L). In Michaelis–Menten models, the two *K*_M_ parameters associated with two substrate reactions were assumed to be independent one from another. [S_1_] and [S_2_] are the substrate concentrations. Literature *K*_M_ values, obtained typically from reaction conditions in which only one substrate was varied, could thus be used. The *V*_max_, *K*_M_, and *K*_eq_ values used are summarized in Table 1. Γ is the mass action ratio. The *K*_eq_ is the corresponding equilibrium constant. The reactions of SiaC and CSS involve large “forward” driving force and so were considered to be irreversible. Thus, their $\frac{\Gamma }{K\text{eq}}=\sim 0$.

**Table 1:** Enzyme parameters important for 3SL synthesis in a one‐pot cascade reaction

Enzyme	Expression yield^a^ (mg/L culture)	Specific activity^b^ (U/mg protein)	*k*_cat_ (s^−1^)	*K*_M_ (mM)	*k*_cat_/*K*_M_ (s^−1^ mM^−1^)	*K*_eq_ (L mol^−1^)	Optimum pH	Additive requirement
NAL	160	Neu5Ac synthesis: 4.2 Neu5Ac cleavage: 10.1	Neu5Ac synthesis: 4.8^c^ Neu5Ac cleavage: 10.1^c^	ManNAc: 160^c^ Pyruvate 19.9^c^ Neu5Ac: 1.8^c^	ManNAc: 0.03^c^ Pyruvate 0.11^c^ Neu5Ac: 5.6^c^	20.7^d^ 24.1^e^	7.0–7.3^c^	–
SiaC	150	23	0.9^f^	ManNAc: 9.4^f^ PEP: 0.25^f^	ManNAc: 0.1^f^ PEP:‐	8.7 × 10^10^ e	7.5^f^ (Kinetic constants measured at pH 8.3)	1 mM divalent cation (MgCl_2_)^f^
CSS	50	36	19^g^	Neu5Ac: 0.11^g^ CTP: 0.05^g^	Neu5Ac: 172^g^ CTP: 1180^g^	–*	8.5^g^ (kinetic constants measured at pH 8.0)	10 mM divalent cation (MgCl_2_)^g^ 0.2 mM sulfhydryl group^g^ (DTT, Cys)
PdST	200	Transfer: 5.2 Hydrolysis: 3.5^h^ Sialidase: 0.02^h^	Transfer: 3^h^	CMP‐Neu5Ac: 1.1^h^ Lactose: 1.5^h^	CMP‐Neu5Ac: 2.7^h^ Lactose: 16^h^	–**	8.0^h^	–

*Note:* NAL (*N*‐acetyl neuraminate lyase from *Lactobacillus plantarum* WCFS1); SiaC (sialic acid synthase from *Neisseria meningitidis*); CSS (CMP‐sialic acid synthetase from *N. meningitidis*); PdST (α2,3‐sialyltransferase from *Pasteurella dagmatis*).

^a^ Expressed in shaking flasks (250 ml medium in 1 L flasks).

^b^ Measured at one‐pot reaction conditions (pH 8.0, 100 mM Tris/HCl, 37°C); *note*: to measure NAL activity, only 20 mM ManNAc was used. This corresponds to a [S]/*K*_M_ ratio of 0.125.

^c^ Sánchez‐Carrón et al. (2011).

^d^ *K*_eq_ from *E. coli* K‐12 (pH 7.5, 30°C, 0.1 M salt) is used as a reference (Groher & Hoelsch, 2012).

^e^ *K*_eq_ calculated at pH 8.0 and 0.025 M salt by the online‐tool eQuilibrator (Flamholz et al., 2012).

^f^ Gunawan et al. (2005).

^g^ Gilbert et al. (1997); He et al. (2011).

^h^ Schmölzer et al. (2013, 2015).

* Reaction assumed to be quasi‐irreversible.

** *K*_eq_ estimated from model fits (see Section 3).

Besides its main transferase activity, the PdST exhibits hydrolase activity towards CMP‐Neu5Ac (Table 1; Figure 1), releasing CMP and Neu5Ac. The CMP‐Neu5Ac hydrolysis occurs in competition with sialyl transfer to lactose. This can affect the yield of sialoside product in enzymatic synthesis (Schmölzer et al., 2013). At low lactose concentrations at around the *K*_M_ (≤1.5 mM), the ratio between hydrolysis and transfer (*R*_h_) was approximately 0.17 (Schmölzer et al., 2015). To model the PdST reaction, we therefore added *R*_h_ and *K*_eq_ as additional fit parameters. The *R*_h_ was assumed constant (not variable with concentration of acceptor substrate). The low “sialidase” activity of PdST (Table 1; Figure 1) supports the notion of a nonzero $\frac{\Gamma }{K\text{eq}}$ for the sialyltransferase reaction. The equations used to describe the cascade reactions are summarized in Online Supporting Information.

#### 2.5.2 Data fitting

Complete data sets, comprising time courses of all independent reactants of the three‐step conversion (ManNAc, Neu5Ac, CTP, CMP‐Neu5Ac, CMP, and 3SL), were fitted simultaneously. Reactants that were not routinely measured and defined from the mass balance (PYR, PEP, pyrophosphate, and lactose) were not included. Data sets for the NAL/CSS/PdST and SiaC/CSS/PdST cascade reactions were fitted together. All fit parameters were thus obtained from a single fitting process. These parameters were *V*_max_ (four enzymes), *R*_h_ (PdST), and *K*_eq_ (PdST transferase reaction; NAL reaction). We chose this approach focusing primarily on *V*_max_ (with *R*_h_ directly linked to *V*_max_) for its immediate relevance to inform conversion experiments regarding the [E] to be used. Moreover, although change in reaction conditions between assays and synthesis can affect all parameters (*V*_max_, *K*_eq_, and *K*_M_), it is mostly the *V*_max_ that governs the conversion rate in a cascade (see the Section 3.3 on the *K*_eq_ of NAL later).

The fitting used MATLAB's *lsqnonlin* (nonlinear least‐square method with trust‐region‐reflective algorithm) utility. In total, 5000 independent fits were performed. Each fit started by selecting random starting values for each parameter from individually predefined sets, containing 50 values and designed as follows. *V*_max_ starting values were evenly distributed in the range ±50% of the *V*_max_ from literature or the *V*_max_ determined with the herein used assays (Table 1). *R*_h_ starting values were evenly distributed between 0 and 0.1 (Schmölzer et al., 2015). Starting values for *K*_eq_ were logarithmically distributed over three orders of magnitude for the NAL (range 10^−1^ to 10^1^) and the PdST reaction (range 10^0^–10^2^).

Iterative fitting continued until the step size of the objective function fell below step size tolerance (MATLAB default: 10^−6^). The number of used iterations never reached the MATLAB default maximum of 400. The top 5% of the obtained fit parameter combinations based on residual error were further analyzed. After check for plausible agreement with the experimentally determined parameter (Table 1), the median of each series of estimates was used for optimization.

#### 2.5.3 Model guided optimization

The objective was to minimize the total protein loading from a suitable combination of the three enzymes for a given task in the conversion, defined for the NAL and the SiaC cascade under the Section 3.3. A large set of reaction (≥10^5^) conditions was screened computationally using reaction time‐course simulations. For each enzyme used, variation in [E] (mg/L) was constrained to a plausible range informed by preliminary experiments and simulations: NAL, 50–200; SiaC, 5–50; CSS, 5–40; PdST, 20–120. Simulated reactions giving 3SL in a 10% range of the reference experiment were sampled and analyzed further for actual conversion (rounded to one decimal place), total enzyme used, and enzyme ratio. Eventually, the total turnover number (*TTN*; g 3SL/g total protein) was used for selection and two experiments (one for verification, the other for optimization) were derived from the simulations. The verification experiment used a random combination of [E] values with the only requirement that it fulfilled the initially described conversion criterion. The optimization experiment was designed using the median [E] of the top 0.5% most optimized in silico experiments as the basis. The level of top 0.5% was selected considering limits of precision in analyte and protein quantification. All chosen [E] combinations are summarized in Table S2.

## 3 RESULTS AND DISCUSSION

### 3.1 Enzyme characterization for use in cascade reactions

Essential requirement for the planned engineering analysis was that the enzyme preparations used were well defined and characterized. All enzymes were obtained reproducibly (*N* ≥ 5) in high purity (Figure S1) and good to excellent yields (50–200 mg/L culture; Table 1). As known from literature, all enzymes worked well in the pH range 7.0–8.5 (Table 1). Considering that stability of CMP‐Neu5Ac (the most labile among the reactants of the cascade reaction) was best in the pH range 8.0–11.00 (Beau et al., 1984), we chose a “working pH” of 8.0. The CSS reaction requires Mg^2+^. We added 20 mM MgCl_2_ in combination with 25 mM CTP as the substrate. A higher concentration of Mg^2+^ (tested up to 40 mM with 25 mM CTP) neither enhanced the enzymatic rate nor did it improve the CMP‐Neu5Ac yield. We also showed that in coupled reactions of SiaC (20 mM PEP) and CSS (25 mM CTP) no more than 20 mM Mg^2+^ were necessary for maximum conversion rate and product yield. l‐Cysteine (0.2 mM) was added for CSS activity and stability. In our hands, the l‐cysteine was equally effective as the dithiothreitol used in the literature (Mizanur & Pohl, 2008). Stability of 3SL was also examined under reaction conditions in the absence of enzymes (37°C; 100 mM Tris/HCl, pH 8.0, 20 mM MgCl_2_, 0.2 mM Cys; agitation at 450 rpm). Using 10 mM 3SL, no degradation of the compound was detected with HPLC and TLC in 1 h of incubation. Activity assays used the temperature and the pH of the cascade reaction and the substrate concentrations were adapted to the expected accumulation of intermediates during the conversion. Therefore, 5 mM Neu5Ac was used for CSS and 1 mM CMP‐Neu5Ac for PdST. Specific activities recorded under these conditions are summarized in Table 1.

As reference for optimization with each cascade system, a “common sense” synthesis reaction was designed in which equal volumetric activities (0.6 U/ml) of all enzymes were used. ManNAc and lactose were supplied at 20 mM each. PYR was used at 50 mM, following notion from earlier studies that 2.5‐fold molar excess of PYR over ManNAc can drive the Neu5Ac formation (Yu & Chen, 2006). CTP was used in slight excess over ManNAc (25 mM), to account for CMP‐Neu5Ac hydrolyzed by the PdST (Schmölzer et al., 2013). We estimated that the overall conversion (ManNAc → 3SL) proceeding at maximum rate (0.6 mM/min) in each enzymatic step would require approximately 100 min (= 20 × 3/0.6) to complete. Reactions were analyzed for 4 h.

### 3.2 Reactant quantification for time‐course analysis

Modeling of cascade reactions is best supported by analytical quantification of all involved, process‐relevant reactants. Despite the widespread use of the NAL/CSS/PdST cascade reaction for sialo‐oligosaccharide synthesis (Malekan et al., 2013; Tasnima et al., 2019; Yu et al., 2009, 2011), the reaction analysis was typically restricted to product, acceptor substrate and sometimes both (Lau et al., 2011; Yu et al., 2016). Intermediates were not determined. We here therefore set out to quantify ManNAc, Neu5Ac, CTP/CMP, CMP‐Neu5Ac, and 3SL. Based on close mass balance confirmed experimentally, PYR, PEP, lactose, and pyrophosphate were considered redundant and not measured routinely. Using TLC for preliminary assessment of the conversion (Figure S2), we developed HPLC analytical protocols to determine CMP‐Neu5Ac and nucleotides separate from the carbohydrates. In each protocol, a dedicated sample preparation was important to optimally suit the subsequent HPLC analysis. Ion‐pairing on reversed C‐18 stationary phase (nucleotides) or ligand exchange (carbohydrates) was used for separation (Figure 2). To avoid overlap between CTP and 3SL in elution (Figure S3), the nucleotides were hydrolyzed by a phosphatase before carbohydrate analysis. We noted that due to baseline shift caused, the acetonitrile used for enzyme inactivation interfered with the carbohydrate analysis. Heat inactivation was used instead. The analytical procedures were applicable to NAL/CSS/PdST as well as SiaC/CSS/PdST cascade reactions.

### 3.3 SiaC and NAL cascade reactions for 3SL synthesis

**Figure 3:**
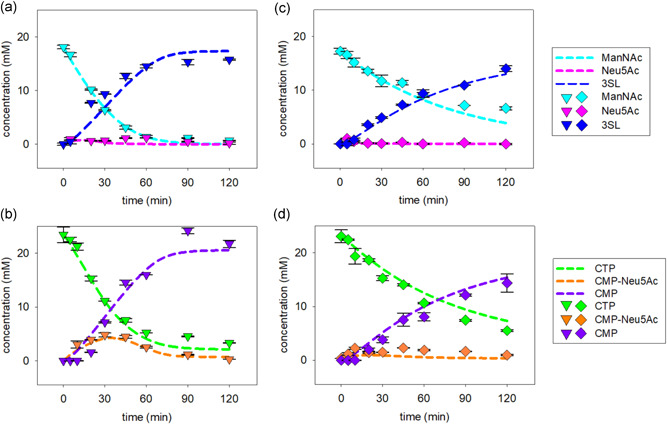
Time courses of SiaC (panels (a) and (b) and NAL (panels (c) and (d)) cascade reactions performed under the reference conditions. Symbols (SiaC, triangles; NAL, diamonds) show the data and the lines are model fits. The reference conditions involved the same loading of each enzyme (0.6 U/ml). CMP, cytidine 5ʹ‐monophosphate; CTP, cytidine 5ʹ‐triphosphate; NAL, Neu5Ac lyase; Neu5Ac, *N*‐acetyl‐d‐neuraminic acid; SiaC, sialic acid synthase

Full‐time courses of 3SL synthesis by SiaC/CSS/PdST and NAL/CSS/PdST reactions were determined (Figure 3). Use of each enzyme at 0.6 U/ml was the basis for comparison. The SiaC reaction (Figure 3a,b) showed a 3SL release that was largely linear with time up to approximately 60 min, corresponding to approximately 90% and approximately 80% conversion of ManNAc and CTP, respectively. The time for conversion (∼100 min) was as estimated from the enzyme activity added. Neu5Ac did not accumulate in the reaction. CMP‐Neu5Ac passed through a kinetic maximum of ∼5 mM after around 40 min, only to decrease later in the reaction. From a comparison of the dynamics of Neu5Ac, CMP‐Neu5Ac, and 3SL (Figure 3a,b), we concluded that the CSS reaction proceeded relatively faster than the SiaC and PdST reactions. The mean molar ratio of CMP/3SL was ∼1.2:1. The result suggested ∼15% hydrolysis of CMP‐Neu5Ac. The composition of the reaction mixture did not change appreciably (±0.5 mM) upon longer incubation up to 4 h (data not shown). While the low sialidase activity of the PdST (reverse sialyl transferase in combination with CMP‐Neu5Ac hydrolase activity) can hydrolyze 3SL, the released Neu5Ac can be recycled to CMP‐Neu5Ac by CSS, because of the 5 mM excess CTP present in the reaction. Therefore, we were not able to see any loss of product in 4 h of reaction. The maximum 3SL yield (= mM 3SL/mM of limiting substrate) was 82 (±6) % (10.4 ± 0.62 g/L), based on the ManNAc supplied. The ManNAc was converted more fully (∼95%) and material from it was distributed into low residual amounts of Neu5Ac (and CMP‐Neu5Ac). Approximately 90% of the initial CTP was used and released as CMP. The concentrations of all substrates, intermediates and (side) products after 2 h of reaction are summarized in Table S4.

The NAL reaction (Figure 3c,d) was overall slower (∼2‐fold) than the SiaC reaction. The 3SL release rates in the initial 20 min were 7 g/(L h) and 15 g/(L h), respectively. The 3SL time‐course featured a substantial decrease in the production rate already at low degrees of substrate conversion (≤ 50%; 45–60 min). Neu5Ac did not accumulate and CMP‐Neu5Ac did so only slightly. From these reactant profiles, the NAL reaction appeared to have been limiting overall. The 3SL yield after 2 h was 70 ± 3% (8.7 ± 0.26 g/L). The molar ratio of CMP/3SL was approximately 1.2:1, at the end, like in the SiaC reaction.

To exclude that enzyme inactivation could have restricted the attainable degree of substrate conversion and the 3SL yield, we measured the stability of each enzyme under the bulk conditions of the reaction in the absence of substrates. As shown in Table S3, activity loss was negligible over 1 h. Additionally, we analyzed samples taken directly from the reaction after 2 h. There was only a minor decrease in the activity (≤ 20%) of the enzymes (Table S5).

### 3.4 Modeling of the enzymatic cascade reactions

**Figure 4:**
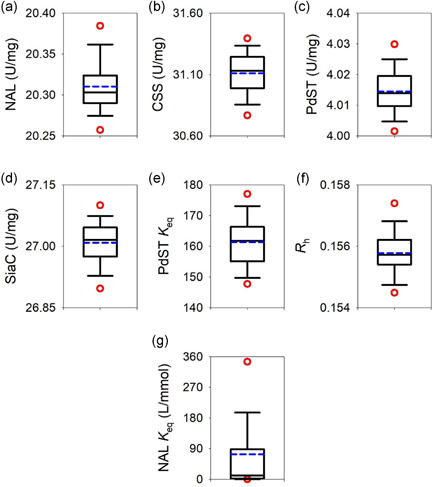
Boxplots of the fitted parameters derived from the top 5% simulations based on the sum of residual errors. The median is indicated by a black line, while the mean is shown in color and boxes extend from the 25th to the 75th percentile of each group's distribution. Whiskers show the 10th and the 90th percentile, respectively. The 5th and 95th percentiles are plotted as red dots. CSS, cytidine 5ʹ‐monophosphate‐sialic acid synthetase; NAL, Neu5Ac lyase; Neu5Ac, *N*‐acetyl‐d‐neuraminic acid; PdST, a2,3‐sialyltransferase from *P. dagmatis*; SiaC, sialic acid synthase

We used mass‐action kinetics with parameters (*K*_M_) accounting for dependence of the reaction rate on the substrate concentration (Equation 1). As shown in Figure 3, using *K*_M_ values from literature (Table 1) and applying *V*_max_ (including *R*_h_) and *K*_eq_ as the adjustable parameters to fit both cascade reactions simultaneously, the *K*_M_ model gave an excellent and coherent description of the experimental time courses of the SiaC (panels (a) and (b)) and NAL cascade conversions (panels (c) and (d)). Moreover, the *V*_max_ values obtained (Figure 4a–d) were in good agreement with results of the enzyme activity assays (Table 1). The *K*_eq_ for the transferase reaction of PdST (Figure 4e) was in a defined narrow range far on the product side. Its average value of 158 was in accordance with literature (Schmölzer et al., 2013), reporting a ratio of approximately 300 for the enzymatic rates of forward (24 s^−1^) and reverse sialyl transfer (=sialidase activity in the presence of CMP; 0.08 s^−1^) from CMP‐Neu5Ac to lactose. The estimated hydrolysis/transfer coefficient *R*_h_ (Figure 4f) was consistent with earlier results of initial rate analysis (Schmölzer et al., 2015). In contrast to the other parameters (Figure 4), the *K*_eq_ for the NAL reaction (Figure 4g) was not well defined as estimated from fitting. This apparent issue was resolved by understanding that in a cascade reaction, the *K*_eq_ on an intermediate step can be neglected if a constant removal of the product is ensured (Ricca et al., 2011). Figure S4 shows simulated time courses of ManNAc consumption by a hypothetical NAL reaction in which *K*_eq_ and *K*_M_ for ManNAc were variable. The results reveal that variation in the *K*_eq_ was without influence, explainable on account of the “pull” from the effectively irreversible CSS reaction. By contrast, lowering the *K*_M_ for ManNAc resulted in a significant increase the ManNAc consumption rate (Figure S4). The fitting results additionally revealed that in both cascade transformations but especially when SiaC was used (Figure 3a,b), the overall sialidase reaction, that is, the reverse PdST reaction coupled to hydrolysis of the CMP‐Neu5Ac thus formed, contributed to limitation of the 3SL yield after 2 h.

Overall, the fitting result was robust (Figure 3), and the parameter estimates well defined (Figure 4). The median *V*_max_ was calculated to be 20.30 U/mg for NAL (a), 31.13 U/mg for CSS (b), 4.01 U/mg for PdST (c), and 27.02 for SiaC U/mg (d). The median *R*_h_ was 0.155 (f). The median *K*_eq_ was 161.7 for PdST (e) and 11.23 for NAL (g). Note that the apparent median *V*_max_ for PdST was 3.39 U/mg, after accounting for the inherent hydrolase activity. The fit quality for each compound's time‐course was analyzed by calculating the Pearson's correlation coefficient (*R*): ManNAc (NAL: 0.994, SiaC: 0.998), Neu5Ac (NAL: 0.783, SiaC: 0.647), CTP (NAL: 0.995, SiaC: 0.997), CMP‐Neu5Ac (NAL: 0.772, SiaC: 0.978), CMP (NAL: 0.995, SiaC: 0.986), and 3SL (NAL: 0.995, SiaC: 0.955). The description of substrates and products (typical error for 3SL ≤ 10%) was generally good, while there was a relatively larger error on the description of intermediates formed at low concentration (Neu5Ac, CMP‐Neu5Ac).

### 3.5 Modeling‐based optimization of the enzyme loading

**Figure 5:**
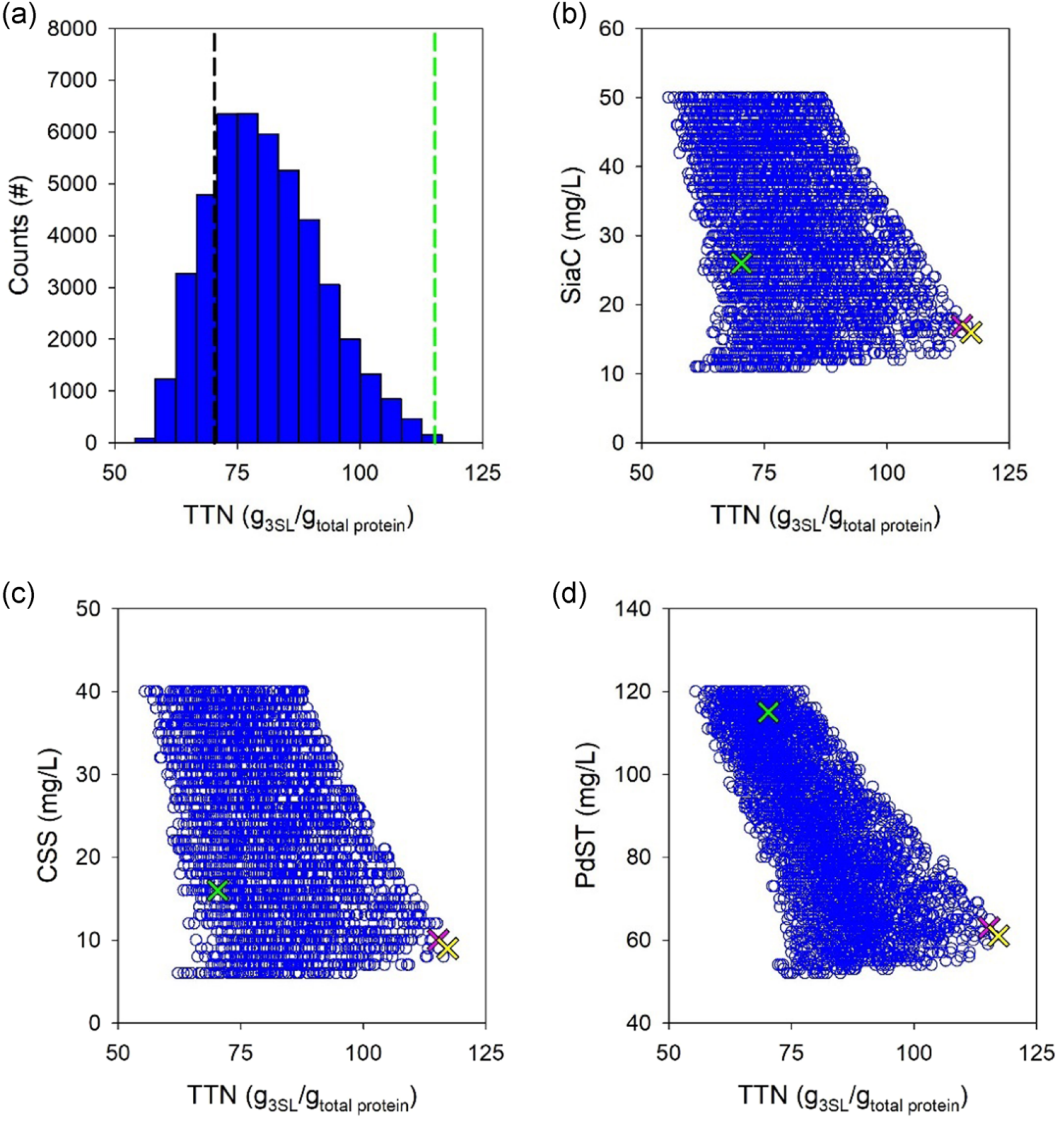
Simulations of the SiaC cascade reaction for window‐of‐operation analysis and optimization. (a) *TTN* distribution of simulated conditions that achieve the required conversion of 17.4 mM 3SL in 2 h. The black line shows the *TTN* of the reference experiment (see Figure 3a,b) and the green line indicates the *TTN* of the optimized reaction. (b)–(d) Use of single enzymes analyzed according to *TTN* achieved, shown for SiaC (b), CSS (c), and PdST (d). Each open blue circle shows an in silico experiment. Note that the *TTN* can vary for a given single enzyme concentration dependent on the concentrations of the two other enzymes. The green, yellow, and magenta crosses show the original, theoretically best and selected [E], respectively. In panels (b)–(d), only every 30th in silico experiment is shown to allow an easier viewing (*N*_total_ = 90,324). CSS, cytidine 5ʹ‐monophosphate‐sialic acid synthetase; PdST, α2,3‐sialyltransferase from *P. dagmatis*; SiaC, sialic acid synthase; *TTN*, total turnover number

**Figure 6:**
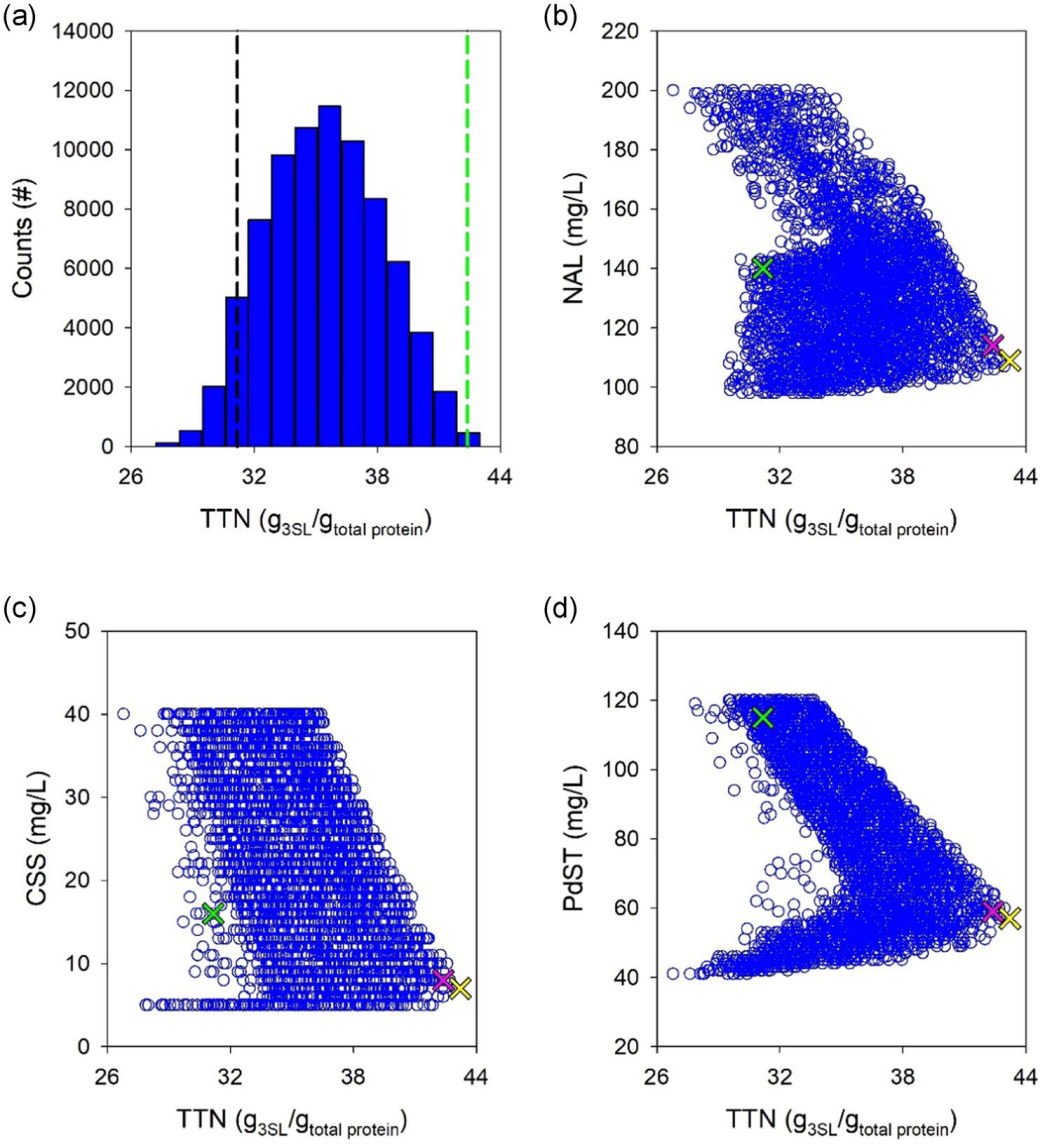
Simulations of the NAL cascade reaction for window‐of‐operation analysis and optimization. (a) *TTN* distribution of simulated conditions that achieve the required conversion of 13.0 mM 3SL in 2 h. The black line shows the *TTN* of the reference experiment (see Figure 3c,d) and the green line indicates the *TTN* of the optimized reaction. (b)–(d) Use of single enzymes analyzed according to *TTN* achieved, shown for NAL (b), CSS (c), and PdST (d). Each open blue circle shows an in silico experiment. Note that the *TTN* can vary for a given single enzyme concentration dependent on the concentrations of the two other enzymes. The green, yellow, and magenta crosses show the original, theoretically best and selected [E], respectively. In panels (b)–(d), only every 30th in silico experiment is shown to allow an easier viewing (*N*_total_ = 156,471). CSS, cytidine 5ʹ‐monophosphate‐sialic acid synthetase; NAL, Neu5Ac lyase; PdST, α2,3‐sialyltransferase from *P. dagmatis; TTN*, total turnover number

**Figure 7:**
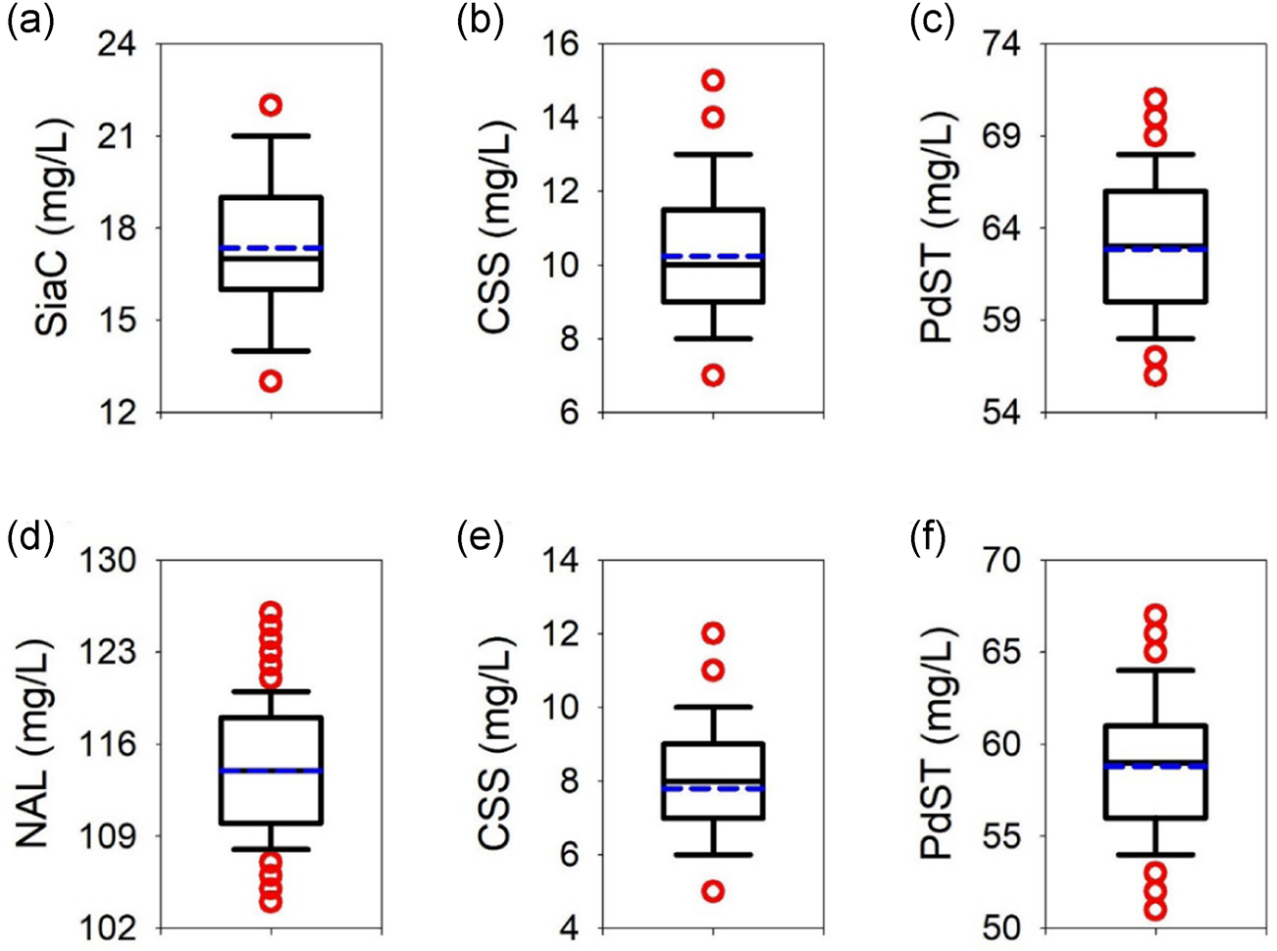
Boxplots of the optimized enzyme concentrations for the SiaC (a)–(c) and NAL (d)–(f) cascade reactions. The median is indicated by a black line while the mean is shown in color and boxes extend from the 25th to the 75th percentile of each group's distribution. Whiskers show the 10th and the 90th percentile, respectively. Data points outside the 10th and 90th percentile are shown as red dots. CSS, cytidine 5ʹ‐monophosphate‐sialic acid synthetase; NAL, Neu5Ac lyase; PdST, α2,3‐sialyltransferase from *P. dagmatis*; SiaC, sialic acid synthase

Objective for the model‐based optimization was minimized total amount of protein used to synthesize 3SL as in the reference experiment (±10% tolerance) in a 2‐h reaction. Thus, the *TTN* (g 3SL/g total protein) would be maximized. Concretely, the 3SL target was 17.4 mM for the SiaC cascade, 13.0 mM for the NAL cascade. For each cascade reaction, computational sampling of conditions reaching the desired conversion was done from a large set of simulated experiments (SiaC: 167254; NAL: 549036). The total distribution of hits according to the *TTN* reached is shown in Figure 5a for the SiaC cascade and in Figure 6a for the NAL cascade. The results reveal significant potential for optimization of the *TTN*. Comparing the reference experiment with the top bin of the calculated distributions, a 1.7‐fold improvement in *TTN* (70 → 120) was suggested for the SiaC cascade (Figure 5a). For the NAL cascade (Figure 6a), the corresponding improvement in *TTN* was 1.4‐fold (31 → 43). The hits were further analyzed according to content of individual enzyme used in the simulated conditions. The individual enzymes varied in a broad range. The CSS showed the largest variation (∼8‐fold) in both cascades (Figures 5c and 6c). The enzyme with the lowest variation was PdST in the SiaC cascade (2.4‐fold; Figure 5d) and NAL in the corresponding cascade (2‐fold; Figure 6b). Figures 5b–d and 6b–d are important for optimization because they immediately suggest, for each enzyme, the operational region for maximum overall *TTN*. To select an optimum point for experimental verification with both cascades, we chose the median [E] of the top 0.5% hits whose distributions are shown Figure 7 (SiaC: panels (a)–(c); NAL: panels (d)–(f)). The experimental conditions are summarized in Table S2.

The optimized SiaC reaction involved a approximately 43% decrease in total enzyme usage (89 mg/L) compared with the reference reaction (156 mg/L). The mass loading (mg/L) for each enzyme was also decreased: SiaC, 26 → 17; CSS, 16 → 10; and PdST, 115 → 63. It is interesting to note that the optimized enzyme loading as regards mass resulted in a balanced ratio of the individual enzyme activities (SiaC: 0.39 U/ml; CSS: 0.38 U/ml; PdST: 0.33 U/ml). This is consistent with the notion that the overall flux through the cascade reaches an optimum when the individual fluxes are balanced. Analysis of cascade reaction efficiency dependent on the PdST loading revealed trade‐off between *TTN* and 3SL yield. Figure S6 shows that for a PdST concentration of below approximately 80 mg/L, the 3SL yield decreased. The *TTN*, however, showed an opposite trend (Figure 5d) to decreasing above approximately 80 mg/L.

The optimized NAL reaction involved decrease by approximately 33% in total enzyme usage (181 mg/L) compared with 271 mg/L in the reference reaction. The mass loading (mg/L) of the individual enzymes was also decreased: NAL, 140 → 114; CSS, 16 → 8; and PdST, 115 → 59. In terms of activity, CSS (0.30 U/ml) and PdST (0.31 U/ml) were balanced, NAL (0.49 U/ml) was present in 1.6‐fold excess. It can be noted, therefore, that the NAL reaction initially used a [ManNAc]/*K*_M_ ratio of approximately 0.125. This can be compared with the way more advantageous ratio of approximately 2 for the SiaC reaction. Comparison with the SiaC cascade (Figure 5) shows that the NAL cascade involved a narrower “window of operation” for the individual enzymes, in particular PdST.

**Figure 8:**
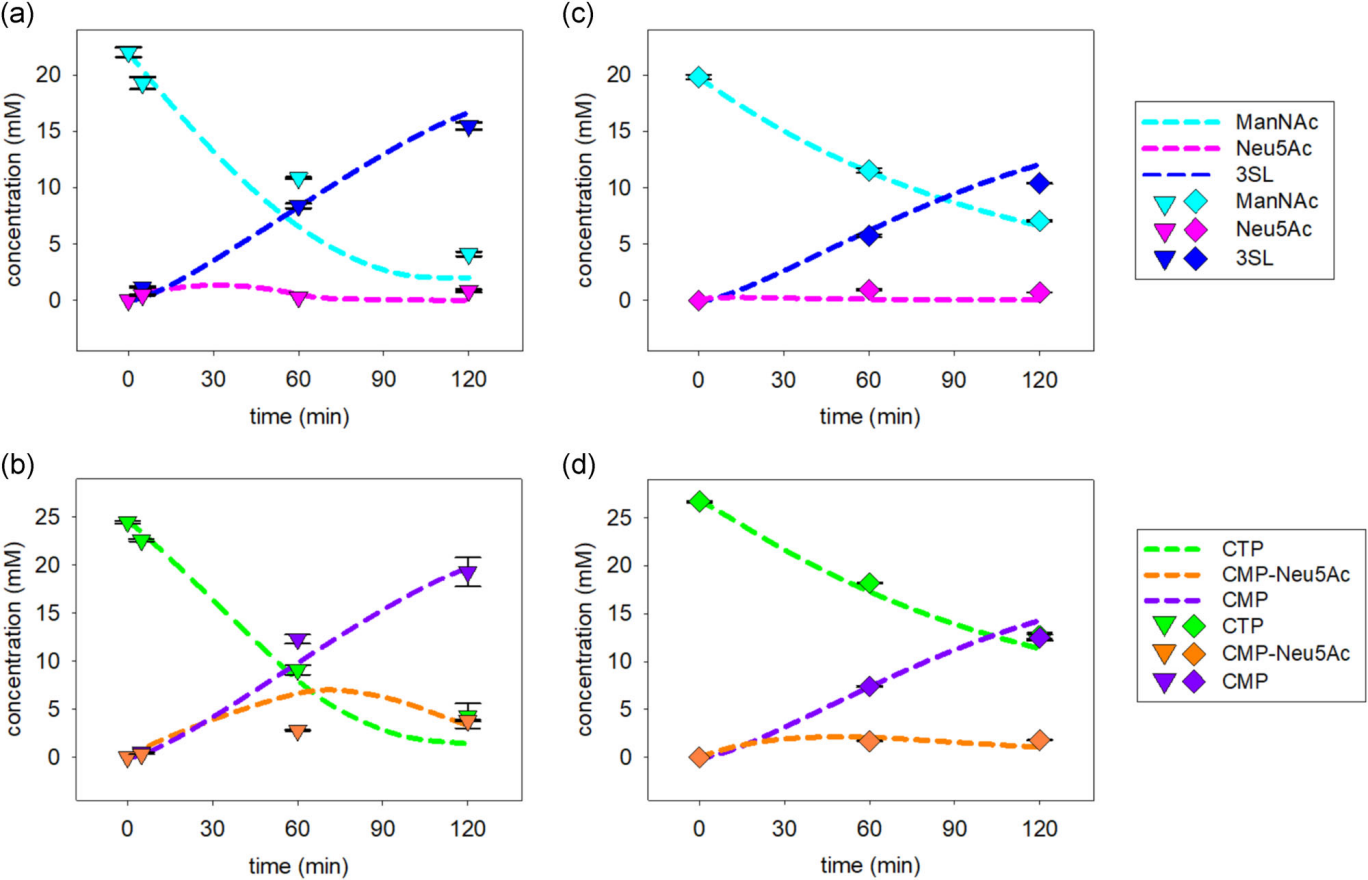
Time courses of SiaC (panels (a) and (b)) and NAL (panels (c) and (d)) cascade reactions performed under the optimized conditions. Symbols (SiaC, triangles; NAL, diamonds) show the data and the lines are model fits. The enzyme concentrations are from Figure 7. 3SL, 3ʹ‐sialyllactose; CMP, cytidine 5ʹ‐monophosphate; CTP, cytidine 5ʹ‐triphosphate; ManNAc, *N*‐acetyl‐d‐mannosamine; NAL, Neu5Ac lyase; Neu5Ac, *N*‐acetyl‐d‐neuraminic acid; SiaC, sialic acid synthase

Experimental results from the optimized SiaC and NAL reactions are shown in Figure 8. The NAL reaction showed excellent agreement with the model predictions at the level of all reactants (substrates, intermediates, and products) involved. The SiaC reaction was generally very well in accordance with the model predictions, except for the intermediary concentrations of ManNAc (panel (a)) and CMP‐Neu5Ac (panel (b)) at 60 min. Duplicate experiments confirmed the data and measurements done at shorter (5 min) and longer times (120 min) were in good agreement with the model (Figure 8a,b). Curiously enough, the reference reaction (Figure 3a,b) and a nonoptimized verification reaction (Figure S5) described below did not show similar deviation between model and experiment. The results appear to imply that the SiaC reaction under the conditions optimized for enzyme loading (Figure 8a,b) was slower than expected from the model. Although used in metabolic engineering for whole‐cell production of 3SL (Drouillard et al., 2010; Fierfort & Samain, 2008), the SiaC was not well studied as regards its kinetic properties and the possible inhibition by metabolites. Detailed characterization of the SiaC to expand the thermodynamic‐kinetic model of the three‐enzyme cascade reaction for improved capture of the intermediates' dynamics was beyond the goals of the current inquiry. At this stage, we considered the applied model to represent a usefully accurate engineering tool with which to pursue optimization tasks.

However, to further support the model, we selected for each cascade a random operational point (SiaC: Figure 5b–d; NAL: Figure 6b–d) within the region previously identified from simulations. The exact conditions are summarized in Table S2 and the results are shown in Figure S5. The excellent agreement between measured and simulated time‐course data for both cascade reactions was strong evidence for model verification.

## 4 CONCLUSIONS

Modeling‐based approach to the optimization of three‐enzyme cascade reactions for 3SL synthesis was presented. Besides the NAL cascade known from earlier studies (Schelch et al., 2020), the SiaC cascade was used here for the first time in bio‐transformations in vitro. The optimization strategy was innovative: it was built on time‐course simulations with a mass action‐controlled kinetic model that were done in substantial computational bulk (≥10^5^ conditions) to screen a large, rationally defined operational space. The simulation results obtained thus enabled important optimization tasks to be analyzed flexibly. This was demonstrated for maximized *TTN*, based on individually optimized enzyme loadings, to reach a predefined conversion target. The optimized SiaC cascade gave higher 3SL yields (79% compared with 65%) and productivity (2‐fold) than the optimized NAL cascade, and its corresponding *TTN* was almost three‐fold higher. Comparison can be done with literature at the level of similar product concentration formed (Table S5). This shows that the computationally identified and experimentally verified conditions of optimized 3SL synthesis represented improvement by at least one magnitude order for the *TTN*, the productivity or both. The engineering analysis shown here can be generally relevant to promote the field of systems bio‐catalysis (Fessner, 2015; France et al., 2017; Schmidt‐Dannert & Lopez‐Gallego, 2016; Yang et al., 2019), working with multienzyme cascade reactions in vitro (Lau et al., 2011; Malekan et al., 2013; Tasnima et al., 2019; Yu & Chen, 2016) but also in context of whole‐cell metabolism (Faijes et al., 2019; Lu et al., 2021; Sprenger et al., 2017). It can be important to unlock the full potential of glycosyltransferase cascade reactions (Li et al., 2019; Mestrom et al., 2019; Nidetzky et al., 2018; Schelch et al., 2020; Yu & Chen, 2016) for efficient use in oligosaccharide and glycoside production. Lastly, it can support the making of fundamental choices in the development of enzyme cascade transformations, in particular which reactions should be telescoped in one pot and which rather not (e.g., Klermund et al., 2017; Rexer et al., 2020). The NAL cascade provides an interesting example for it could involve synthesis of Neu5Ac (Kragl et al., 1991; Lin et al., 2013; Lv et al., 2017; Schmideder et al., 2017; Tao et al., 2011) spatiotemporally separated from the sialoside formation, or integrated with it as shown here.

## Supporting information

Supporting information.

## Data Availability

The data that support the findings of this study are available from the corresponding author upon reasonable request.
