# Supplementary material for: Engineering analysis of multienzyme cascade reactions for 3ʹ‐sialyllactose synthesis
Source: Biotechnol Bioeng. 2021 Aug 2;118(11):4290–304. doi: 10.1002/bit.27898 (PMC9290085; doi:10.1002/bit.27898)
Supplement: Supplementary file 1 — Supporting information. [file BIT-118-4290-s001.docx]

**SUPPORTING INFORMATION**

**Engineering analysis of multienzyme cascade reactions for 3'-sialyllactose synthesis**

Sabine SCHELCH^a,b^, Manuel EIBINGER^b^, Stefanie GROSS BELDUMA^b^, Barbara PETSCHACHER^a,b^, Jürgen KUBALLA^c^, and Bernd NIDETZKY^a,b,^*

^a^ Austrian Centre of Industrial Biotechnology, Krenngasse 37, A-8010 Graz, Austria

^b^ Institute of Biotechnology and Biochemical Engineering, Graz University of Technology, NAWI Graz, Petersgasse 12, A-8010 Graz, Austria

^c^ GALAB Laboratories GmbH, Am Schleusengraben 7, D-21029 Hamburg, Germany

*Corresponding author: Bernd Nidetzky, e-mail: bernd.nidetzky@tugraz.at, phone: +43 316 873 8400

**MODEL EQUATIONS**Reactions for NAL, SiaC, CSS and PdST were described by Michaelis-Menten parameter (*V*_max_, *K*_M_) mass action kinetics, as shown in Eq. (S1-S4). The mass action ratio (Γ) is defined for NAL and PdST in Eq. (S5) and Eq. (S6), respectively.
The mass balance for all modeled reactants is shown in Eq. (S7-S14) and Eq. (S15-S22) for the SiaC and NAL cascades, respectively.
Eq. (S12-S13) and Eq. (S21-S22) are modified with the term (1-*R*_h_) to account for hydrolysis of CMP-Neu5Ac by PdST. In Eq. (S9) and Eq. (S17), the term V(PdST)*R*_h_ accounts for the released Neu5Ac from CMP-Neu5Ac hydrolysis. Note: Equations describing CMP-Neu5Ac consumption (Eq. S11 and Eq. S19) and CMP production (Eq. S14 and Eq. S22) are not modified by *R*_h_ or (1-*R*_h_). The CMP-Neu5Ac is consumed, and the CMP is released, by sialyl transfer as well as by hydrolysis.

**Michaelis-Menten mass action kinetics**

$$\begin{aligned} V\left( NAL \right)=\frac{V_{\max\left( NAL \right)}\left[ NAL \right]\left[ ManNAc \right]\left[ PYR \right]}{\left( K_{M\left( ManNAc \right)}+\left[ ManNAc \right] \right)\left( K_{M\left( PYR \right)}+\left[ PYR \right] \right)}\left( 1-\frac{\Gamma_{\left( NAL \right)}}{K_{eq\left( NAL \right)}} \right)\#Eq.\left( S1 \right) \end{aligned}$$

$$\begin{aligned} V\left( SiaC \right)=\frac{V_{\max\left( SiaC \right)}\left[ SiaC \right]\left[ ManNAc \right]\left[ PEP \right]}{\left( K_{M\left( ManNAc \right)}+\left[ ManNAc \right] \right)\left( K_{M\left( PEP \right)}+\left[ PEP \right] \right)}\#Eq.\left( S2 \right) \end{aligned}$$

$$\begin{aligned} V\left( CSS \right)=\frac{V_{\max\left( CSS \right)}\left[ CSS \right]\left[ Neu5Ac \right]\left[ CTP \right]}{\left( K_{M\left( Neu5Ac \right)}+\left[ Neu5Ac \right] \right)\left( K_{M\left( CTP \right)}+\left[ CTP \right] \right)}\#Eq.\left( S3 \right) \end{aligned}$$

$$\begin{aligned} V\left( PdST \right)=\frac{V_{\max\left( PdST \right)}\left[ PdST \right]\left[ CMP5NeuAc \right]\left[ Lactose \right]}{\left( K_{M\left( CMP5NeuAc \right)}+\left[ CMP5NeuAc \right] \right)\left( K_{M\left( Lactose \right)}+\left[ Lactose \right] \right)}\left( 1-\frac{\Gamma_{\left( PdST \right)}}{K_{eq\left( PdST \right)}} \right)\#Eq.\left( S4 \right) \end{aligned}$$

$$\begin{aligned} \Gamma_{\left( NAL \right)}=\frac{\left[ Neu5Ac \right]}{\left[ ManNAc \right]\left[ PYR \right]}\#Eq.\left( S5 \right) \end{aligned}$$

$$\begin{aligned} \Gamma_{\left( PdST \right)}=\frac{\left[ 3SL \right]\left[ CMP \right]}{\left[ CMPNeu5Ac \right]\left[ Lactose \right]}\#Eq.\left( S6 \right) \end{aligned}$$

**SiaC cascade reaction**

$$\begin{aligned} \frac{d\left[ ManNAc \right]}{dt}=-V\left( SiaC \right)\#Eq.\left( S7 \right) \end{aligned}$$

$$\begin{aligned} \frac{d\left[ PEP \right]}{dt}=-V\left( SiaC \right)\#Eq.\left( S8 \right) \end{aligned}$$

$$\begin{aligned} \frac{d\left[ Neu5Ac \right]}{dt}=V\left( SiaC \right)-V\left( CSS \right)+V\left( PdST \right)R_{h}\#Eq.\left( S9 \right) \end{aligned}$$

$$\begin{aligned} \frac{d\left[ CTP \right]}{dt}=-V\left( CSS \right)\#Eq.\left( S10 \right) \end{aligned}$$

$$\begin{aligned} \frac{d\left[ CMPNeu5Ac \right]}{dt}=V\left( CSS \right)-V\left( PdST \right)\#Eq.\left( S11 \right) \end{aligned}$$

$$\begin{aligned} \frac{d\left[ Lactose \right]}{dt}=-V\left( PdST \right)\left( 1-R_{h} \right)\#Eq.\left( S12 \right) \end{aligned}$$

$$\begin{aligned} \frac{d\left[ 3SL \right]}{dt}=V\left( PdST \right)\left( 1-R_{h} \right)\#Eq.\left( S13 \right) \end{aligned}$$

$$\begin{aligned} \frac{d\left[ CMP \right]}{dt}=V\left( PdST \right)\#Eq.\left( S14 \right) \end{aligned}$$

**NAL cascade reaction**

$$\begin{aligned} \frac{d\left[ ManNAc \right]}{dt}=-V\left( NAL \right)\#Eq.\left( S15 \right) \end{aligned}$$

$$\begin{aligned} \frac{d\left[ PYR \right]}{dt}=-V\left( NAL \right)\#Eq.\left( S16 \right) \end{aligned}$$

$$\begin{aligned} \frac{d\left[ Neu5Ac \right]}{dt}=V\left( NAL \right)-V\left( CSS \right)+V\left( PdST \right)R_{h}\#Eq.\left( S17 \right) \end{aligned}$$

$$\begin{aligned} \frac{d\left[ CTP \right]}{dt}=-V\left( CSS \right)\#Eq.\left( S18 \right) \end{aligned}$$

$$\begin{aligned} \frac{d\left[ CMPNeu5Ac \right]}{dt}=V\left( CSS \right)-V\left( PdST \right)\#Eq.\left( S19 \right) \end{aligned}$$

$$\begin{aligned} \frac{d\left[ Lactose \right]}{dt}=-V\left( PdST \right)\left( 1-R_{h} \right)\#Eq.\left( S20 \right) \end{aligned}$$

$$\begin{aligned} \frac{d\left[ 3SL \right]}{dt}=V\left( PdST \right)\left( 1-R_{h} \right)\#Eq.\left( S21 \right) \end{aligned}$$

$$\begin{aligned} \frac{d\left[ CMP \right]}{dt}=V\left( PdST \right)\#Eq.\left( S22 \right) \end{aligned}$$

**SUPPORTING METHODS**

**Cloning of SiaC and CSS into the expression vector pC21e1**
Cloning was performed using circular polymerase extension cloning (CPEC; Quan & Tian, 2011). Q5® High-Fidelity DNA polymerase (New England Biolabs, Ipswich, MA, USA) was used for amplification of insert and backbone as well as for the final CPEC assembly. Table S1 shows the sequences of the oligonucleotide primers used for amplification of insert (genes for SiaC and CSS) and backbone (pC21e1). The amplification program used was recommended for Q5^®^ polymerase (see https://www.neb.com). Annealing was performed at 55°C for 30 s and extension at 72°C for 3 min (SiaC) or 2 min (CSS). The final vectors pC21e1_SiaC and pC21e1_CSS were verified by sequencing.

**Enzyme purification via His-tag affinity chromatography**

Cell pellets were resuspended in binding buffer (30 mM sodium phosphate, 300 mM NaCl, 15 mM imidazole, pH 7.4) and disrupted by ultrasonication (6 min in total, alternating 2 s pulse on/4 s pulse off at 60% amplitude) with a Sonic Dismembrator Model 505 (Fisher Scientific, Vienna, Austria). The cell lysate was cleared by centrifugation and filtration. The protein was bound on a HisTrap HP FF column (GE Healthcare, Munich, Germany) and eluted with elution buffer (30 mM sodium phosphate, 300 mM NaCl, 300 mM imidazole, pH 7.4). The purified proteins were desalted using Vivaspin Turbo 10 kDa cut‐off concentrator tubes (Sartorius Stedim, Vienna, Austria) and Tris/HCl buffer (50 mM, pH 7.5). Enzyme purity was checked by SDS-PAGE (Figure S1). Protein concentration was determined with Roti‐Quant reagent (Roth, Karlsruhe, Germany) using BSA as standard. Enzyme aliquots were stored at −20°C.

**Stability test of individual enzymes under one-pot reaction conditions**Enzyme stability was determined at 37°C and 450 rpm in 100 mM Tris/HCl (pH 8.0). The enzyme concentrations were the same as in the reference experiment (Table S2). At different times (0, 15, 30, 45 and 60 min) samples were taken and the activity assays were performed as in Section 2.2.3 of the main text.
Enzyme stability was also determined directly from the reaction. Sample (180 µl) was taken at 2 h of the reference experiment (see Figure 3 of the main text). The sample was mixed with 20 µl of 10-fold concentrated substrate solution for the enzyme assay. The final assay concentrations were 20 mM ManNAc and 50 mM pyruvate for NAL; 20 mM ManNAc and 20 mM PEP for SiaC; 25 mM CTP and 5 mM Neu5Ac for CSS; and 5 mM CMP-Neu5Ac and 15 mM oNP-Gal for PdST. For the SiaC and CSS assays, 20 mM MgCl_2_ was added to the substrate stock solution. The assays and the sample preparation for analysis were exactly as described in Section 2.2.3 of the main text. To determine the initial activity, the same procedure was performed with freshly thawed enzymes. These enzymes were diluted in 100 mM Tris/HCl (pH 8.0) to the same concentration as used in the reaction. A sample (180 µl) was mixed with 20 µl of substrate solution as described above. All assays were performed in at least duplicates.  **Thin layer chromatography (TLC)**TLC was used for fast, semi-quantitative analysis of the degree of conversion in the enzymatic cascade reactions. After stopping the reaction with heat inactivation (99°C, 15 min) and keeping the sample on ice for 15 min, 1 µl sample from the reaction was spotted on TLC Silica gel 60 F_254_ plates (Merck, Darmstadt, Germany). Compounds were separated with 1-butanol:acetic acid:water (2:1:1, by volume) as the solvent and stained with thymol solution. The staining solution contained 0.5% thymol (w/v) in ethanol and sulfuric acid (97%) in a weight ratio of 95:5.

**SUPPORTING TABLES**

Table S1. Sequences of oligonucleotides for amplification of insert and backbone in circular polymerase extension cloning of pC21e1_SiaC and pC21e1_CSS.

| Name | Sequence (5´ → 3´) |
| --- | --- |
| BBpC21e1_SiaC_Fwd | CAAAAAAACTGATATTGAATAAAAGCTTAGG  CATCAAATAAAACG |
| BBpC21e1_SiaC_Fwd | GGTGATGCGATCCTCTCATAGTTAATTTCTCC  TCTTTAATGAATTC |
| SiaC_insert_Fwd | AGGAGAAATTAACTATGAGAGGATCGCATCA  CCATCAC |
| SiaC_insert_Rev | GATGCCTAAGCTTTTATTCAATATCAGTTTTTT  TGATTTGAGCACCTTTGCG |
| BBpC21e1_CSS_Fwd | CATTCTTAATCACAAGGAATAAAAGCTTAGGC  ATCAAATAAAACGAAAG |
| BBpC21e1_CSS_Fwd | CCGCAATATTTTGTTTTTCCATCCCGTGATGGT  GATGGTGATG |
| CSS_insert_Fwd | CACCATCACCATCACGGGATGGAAAAACAAA  ATATTGCGGTTATA |
| CSS_insert_Rev | GATGCCTAAGCTTTTATTCCTTGTGATTAAGAA  TGTTTTCTGCC |

Table S2: Enzyme concentrations used in enzymatic cascade reactions.

| Enzyme used | NAL | | | SiaC | | |
| --- | --- | --- | --- | --- | --- | --- |
|  | *reference* | *verification* | *optimization* | *reference* | *verification* | *optimization* |
| NAL, mg/L | 140 | 105 | 114 | - | - | - |
| SiaC, mg/L | - | - | - | 26 | 20 | 17 |
| CSS, mg/L | 16 | 19 | 8 | 16 | 11 | 10 |
| PdST, mg/L | 115 | 71 | 59 | 115 | 98 | 63 |
| total, mg/L | 271 | 195 | 181 | 157 | 129 | 89 |

Table S3. Enzyme stability under reaction conditions. The conditions of the reference experiments were used.

| Incubation time | Activity, % | | | |
| --- | --- | --- | --- | --- |
|  | SiaC | NAL | CSS | PdST |
| 0 min | 100 | 100 | 100 | 100 |
| 15 min | 104 | 101 | 88 | 92 |
| 30 min | 103 | 104 | 97 | 105 |
| 60 min | 104 | 98 | 100 | 99 |

Table S4. Concentration of substrates, intermediates and products after 2 h of 3SL production. All values are given in mM.

|  | NAL | SiaC |
| --- | --- | --- |
| ManNAc | 6.6 ± 0.28 | 0.7 ± 0.04 |
| Neu5Ac | 0 ± 0.13 | 0.2 ± 0.24 |
| 3SL | 14 ± 0.56 | 16 ± 0.14 |
| CTP | 5.5 ± 0.15 | 3.3 ± 0.04 |
| CMP-Neu5Ac | 1.0 ± 0.12 | 0.3 ± 0.01 |
| CMP | 14 ± 1.73 | 22 ± 0.63 |

Table S5. Residual enzyme activity after 2 h of 3SL production. Mean values of 3 determinations are shown with their standard errors.

|  | Activity, % |
| --- | --- |
| SiaC | 83 ± 8 |
| CSS | 81 ± 3 |
| PdST | 79 ± 6 |
|  |  |
| NAL | 91 ± 2 |
| CSS | 88 ± 4 |
| PdST | 83 ± 7 |

Table S5: Performance parameters of the cascade reaction for 3SL synthesis under reference conditions and after optimization, compared to select examples from literature.

| Set-up | Enzymes used^1^  [U/L] | Product titer  [g/L] | Yield ^2^  [%] | *STY*  [g/L/h] | *TTN*  [g_product_/  g_enzyme_] | Reference |
| --- | --- | --- | --- | --- | --- | --- |
| purified enzymes one-pot reaction  3 enzymes  reference  NAL | NAL: 600  CSS: 600  SiaT: 600 | 8.7 | 70 | 4.4 | 32 | This study |
| purified enzymes one-pot reaction  3 enzymes  reference  SiaC | SiaC: 600  CSS: 600  SiaT: 600 | 10.4 | 79 | 5.2 | 66 | This study |
| purified enzymes one-pot reaction  3 enzymes  optimization  NAL | NAL: 490  CSS: 300  SiaT: 310 | 7.5 | 59 | 3.7 | 37 | This study |
| purified enzymes one-pot reaction  3 enzymes  optimization  SiaC | SiaC: 390  CSS: 380  SiaT: 330 | 9.7 | 76 | 4.8 | 107 | This study |
| purified enzymes one-pot reaction  3 enzymes | NAL: 600  CSS: 600  SiaT: 300 | 5.9 | 80 | 3 | 10.4 | (Yu, Chokhawala, Huang, & Chen, 2006) |
| purified enzymes fusion protein  one-pot reaction | CSS: 4476  SiaT: 373  fusion protein | 31 | 68 | 0.21 | 83 - 167 | (Gilbert et al., 1998) |
| resting cell + purified enzymes  one-pot reaction in two steps | in cell: NAGE: - NAL: -  purified enzymes: CSS: 100  SiaT; 100 | 10.5-11.0 | 78-82 | 0.58-0.61 | 0.71 -0.74 | (Lv et al., 2017) |
| resting cell  3 types of cells  one-pot reaction | CSS: 1976  SiaT: 37 | 36 | 29.5 | 3 | 0.21 | (Endo, Koizumi, Tabata, & Ozaki, 2000) |
| resting cell  2 types of cells  one-pot reaction | NAL: -  CSS: -  SiaT: - | 11.4 | - | 1.63 | 1.14 | (Nahálka & Pätoprstý, 2009) |

SiaT; sialyltransferase; -, not reported

**SUPPORTING FIGURES**


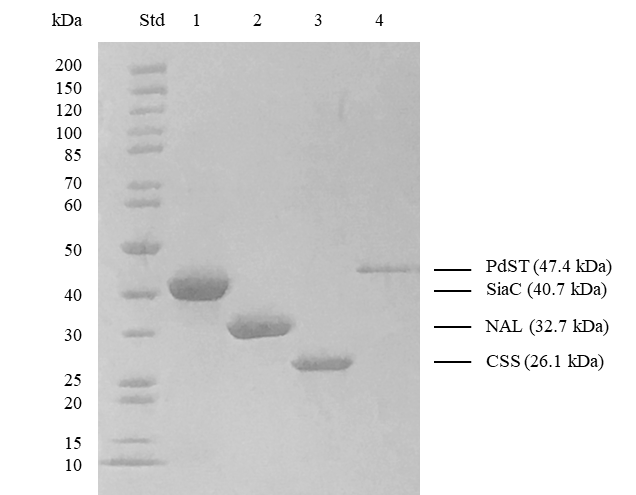


Figure S1. SDS polyacrylamide gel of purified SiaC (1), NAL (2), CSS (3) and PdST (4). Std, molecular mass marker proteins.


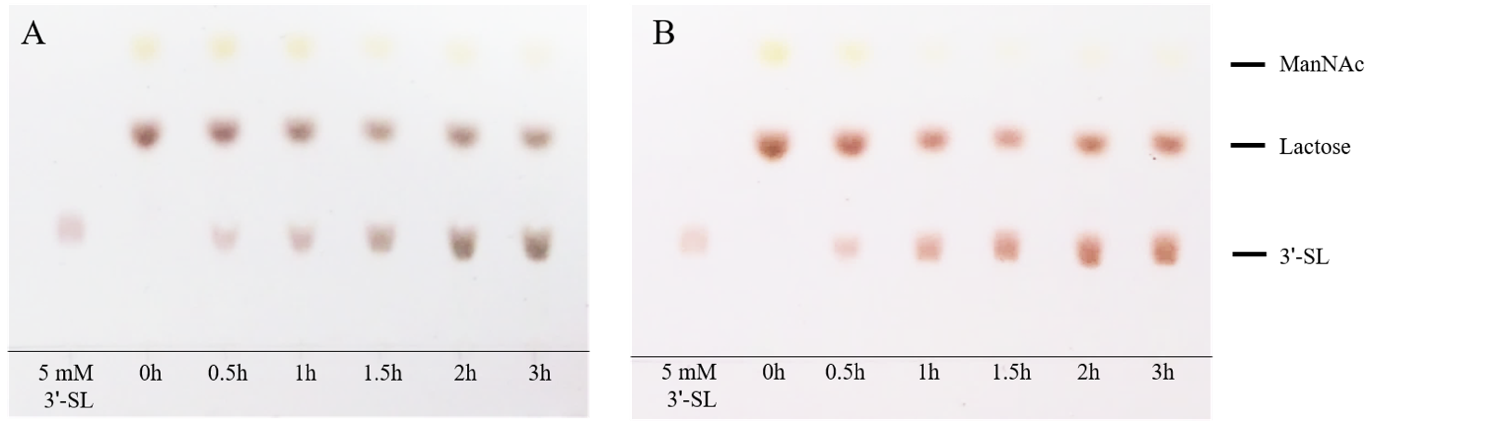


Figure S2. Thin-layer chromatogram of NAL (A) and SiaC (B) cascade reactions.


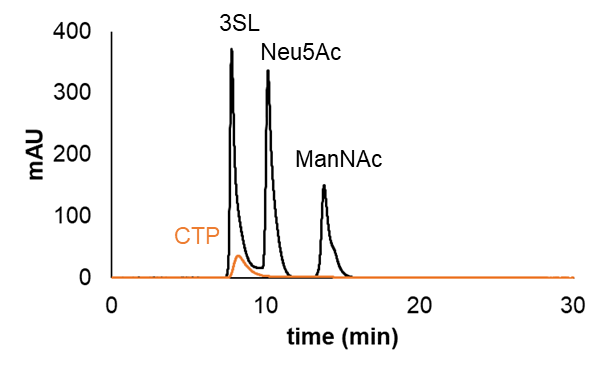


Figure S3: HPLC trace showing 3'-sialyllactose (3SL), *N*-acetyl neuraminic acid (Neu5Ac) and *N*-acetyl mannosamine (ManNAc). CTP (orange) has the same retention time as 3SL and is removed before HPLC analysis by CIP digestion (See SI 1.3).


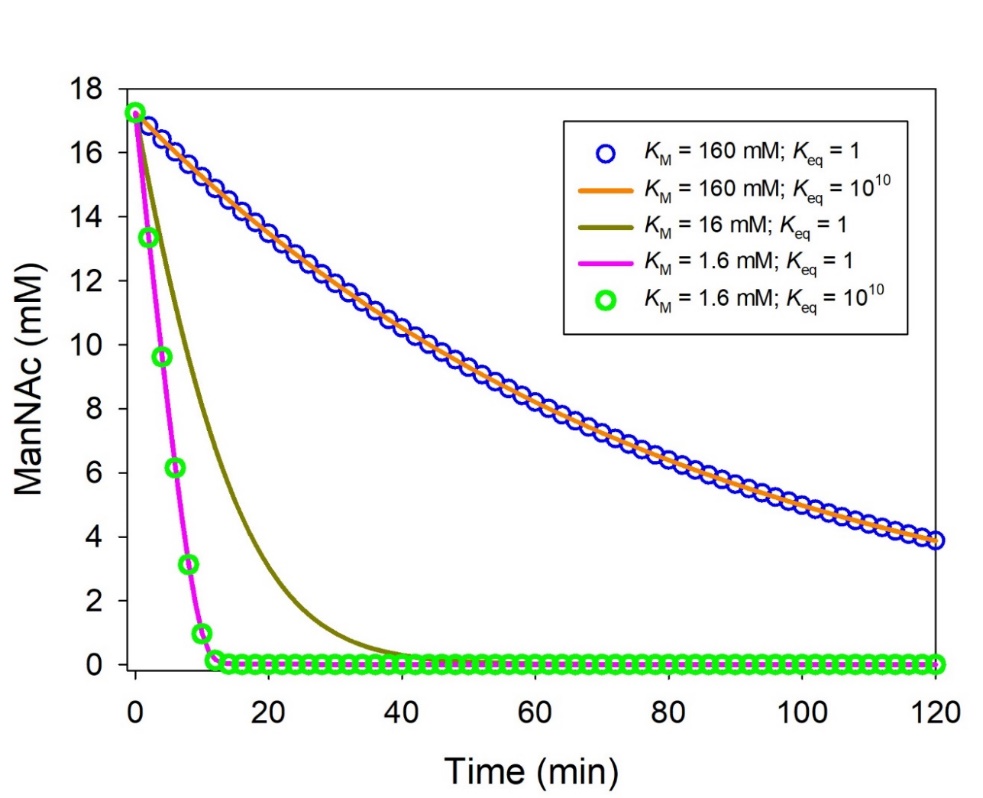


Figure S4: Simulated effect of the NAL *K*_M_, and of the *K*_eq_ of the NAL reaction, on consumption of ManNAc by the NAL cascade reaction. The conditions of the reference experiment were used.


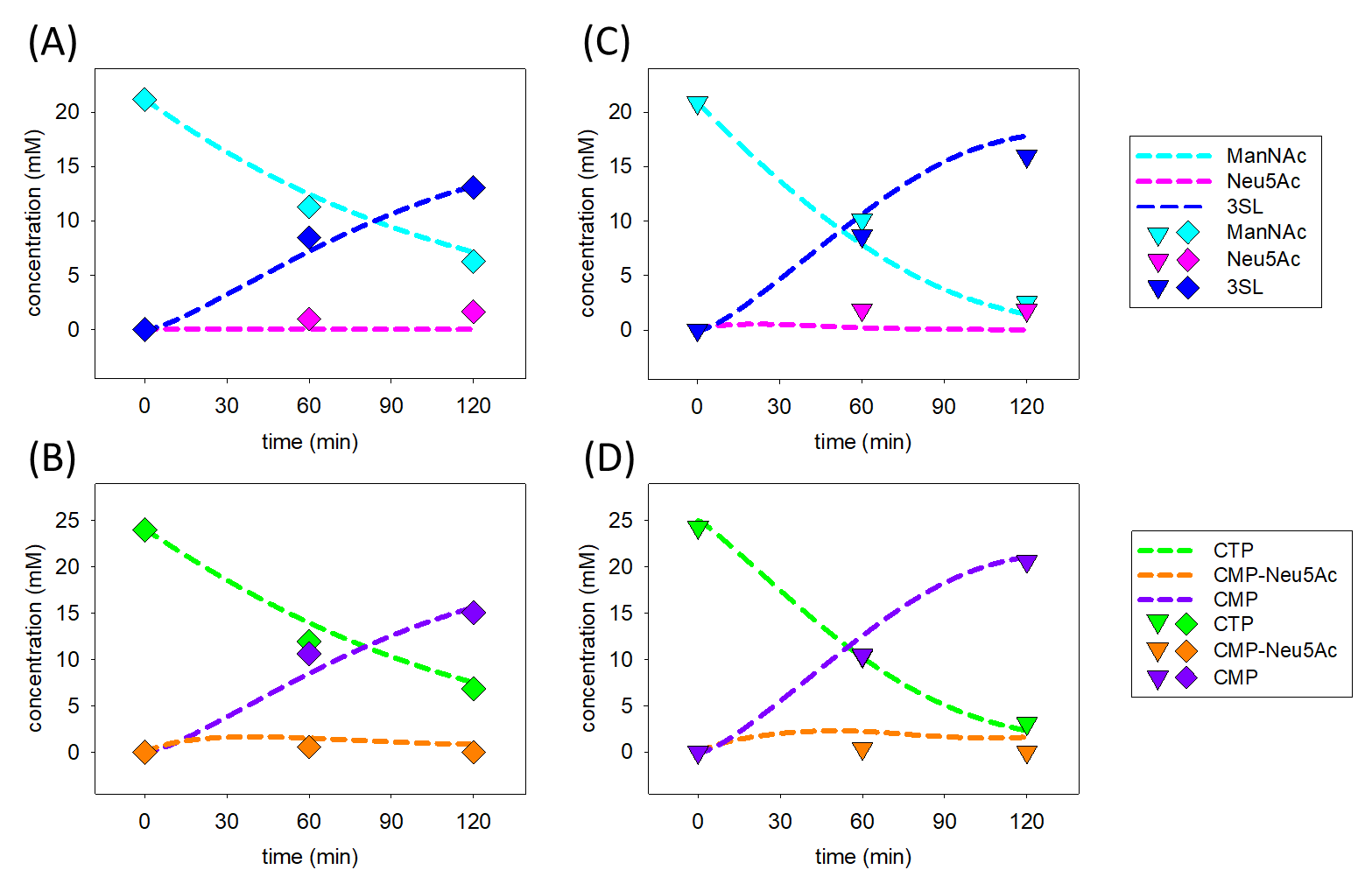


Figure S5: Experiment used for model verification. Comparison of time courses predicted by the kinetic model (lines) and experimental data (NAL, diamonds; SiaC, triangles) of cascade reactions with NAL (A, B) and SiaC (C, D).


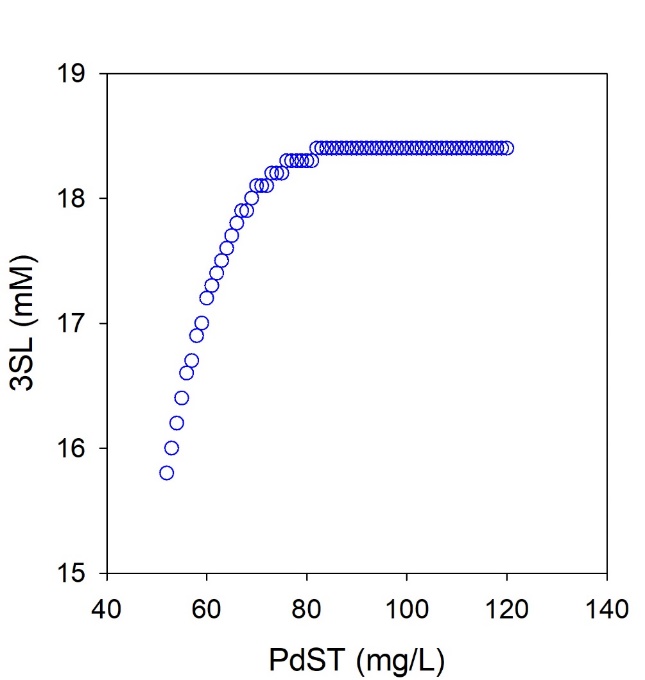


Figure S6. Dependency of 3SL production on the PdST concentration using the SiaC cascade setup. The open blue circles represent the highest possible 3SL concentration for a given PdST concentration. PdST concentrations ≤ 80 mg/L are limiting for the production of 3SL. All simulations achieving the desired conversion (*N*_total_ = 90324) were analyzed independent of their *TTN*.

**REFERENCES**

Endo, T., Koizumi, S., Tabata, K., & Ozaki, A. (2000). Large-scale production of CMP-NeuAc and sialylated oligosaccharides through bacterial coupling. *Applied Microbiology and Biotechnology*, *53*(3), 257–261. https://doi.org/10.1007/s002530050017

Gilbert, M., Bayer, R., Cunningham, A.-M., DeFrees, S., Gao, Y., Watson, D. C., … Wakarchuk, W. W. (1998). The synthesis of sialylated oligosaccharides using a CMP-Neu5Ac synthetase/sialyltransferase fusion. *Nature Biotechnology*, *16*(8), 769–772. https://doi.org/10.1038/nbt0898-769

Lv, X., Cao, H., Lin, B., Wang, W., Zhang, W., Duan, Q., … Li, X. (2017). Synthesis of sialic acids, their derivatives, and analogs by using a whole-cell catalyst. *Chemistry - A European Journal*, *23*(60), 15143–15149. https://doi.org/10.1002/chem.201703083

Nahálka, J., & Pätoprstý, V. (2009). Enzymatic synthesis of sialylation substrates powered by a novel polyphosphate kinase (PPK3). *Organic & Biomolecular Chemistry*, *7*(9), 1778–1780. https://doi.org/10.1039/b822549b

Quan, J., & Tian, J. (2011). PROTOCOL Circular polymerase extension cloning for high- throughput cloning of complex and combinatorial DNA libraries. *Nature Protocols*, *6*(2), 242–251. https://doi.org/10.1007/978-1-62703-764-8_8

Yu, H., Chokhawala, H., Huang, S., & Chen, X. (2006). One-pot three-enzyme chemoenzymatic approach to the synthesis of sialosides containing natural and non-natural functionalities. *Nature Protocols*, *1*(5), 2485–2492. https://doi.org/10.1038/nprot.2006.401
